# Experimental demonstration of pH-dependent electrostatic catalysis of radical reactions†
†Electronic supplementary information (ESI) available: Detailed experimental methods and data. See DOI: 10.1039/c5sc01307k


**DOI:** 10.1039/c5sc01307k

**Published:** 2015-06-23

**Authors:** Marta Klinska, Leesa M. Smith, Ganna Gryn'ova, Martin G. Banwell, Michelle L. Coote

**Affiliations:** a Research School of Chemistry , Australian National University , Canberra ACT 2601 , Australia . Email: michelle.coote@anu.edu.au; b ARC Centre of Excellence for Electromaterials Science , Australia

## Abstract

Time-dependent fluorescence spectroscopy has been used to demonstrate significant pH-dependent electrostatic effects on the kinetics and thermodynamics of hydrogen atom transfer between 1-hydroxy-2,2,6,6-tetramethyl-4-piperidinecarboxylic acid (4-CT-H) and the profluorescent nitroxide {2,2,6,6-tetramethyl-4-[(7-nitro-2,1,3-benzoxadiazol-4-yl)amino]-1-piperidinyl}oxidanyl radical (PFN) in dichloromethane. This pH switching does not occur when 4-CT-H is replaced with a structurally analogous hydroxylamine that lacks an acid-base group, or when the polarity of the solvent is increased. These findings validate our recent theoretical predictions that electrostatic stabilisation of delocalised radicals is of functional significance in low polarity environments.

## Abstract 2

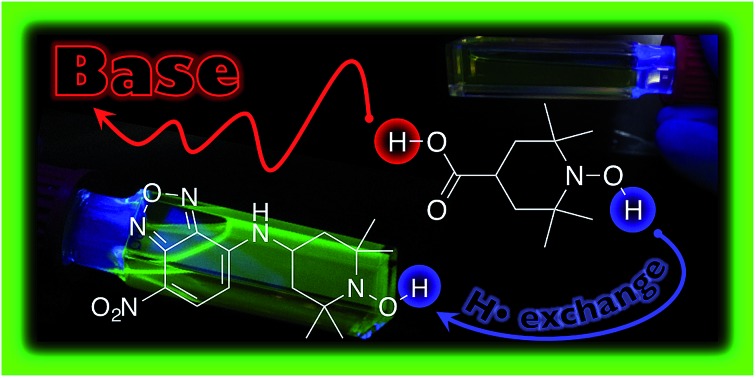
Fluorescence spectroscopy demonstrated pH-dependent electrostatic effects on the kinetics and thermodynamics of hydrogen atom transfer between 1-hydroxy-2,2,6,6-tetramethyl-4-piperidinecarboxylic acid and {2,2,6,6-tetramethyl-4-[(7-nitro-2,1,3-benzoxadiazol-4-yl)amino]-1-piperidinyl}oxidanyl radical in dichloromethane.

## Introduction

Nitroxide (or aminoxyl) radicals, *viz.* R_1_R_2_NO˙ species, are persistent free radicals used in a wide variety of practical applications. While generally resistant to self-termination reactions, they are effective traps for carbon-centred radicals. As such they are utilised as spin traps in kinetic studies, as control agents for free-radical polymerisation^1^ and are the active species when hindered amine light stabilisers are used as radical-trapping antioxidants.^2^ They can also be reversibly oxidised and reduced and are used as reagents in organic synthesis,^3^ as well as superoxide dismutase mimics in medicinal applications.^4^ In addition, they show promise as redox mediators for dye-sensitised solar cells^5^ and organic batteries.^6^ As relatively non-toxic, persistent radicals, they are also useful in a variety of imaging and structure determination applications.^7^

The capacity to manipulate the stability of nitroxide radicals in response to external conditions could greatly enhance their utility. In particular, the ability to tune the stability of nitroxides through simple pH changes is attractive in nitroxide-mediated polymerisation for applications such as sequence control, block copolymer synthesis or end-group exchange. Moreover, if this manipulation of stability could be achieved in such a way as to further *increase* the stability of nitroxide radicals in their “on” state, controlled release of nitroxide radicals and carbon-centred radicals from precursor alkoxyamines could be achieved at much lower temperatures than is possible at the present time. This, in turn, would not only be attractive for nitroxide-mediated polymerisation, but could potentially expand the scope of nitroxides as broadly applicable “radical protecting groups” in chemical synthesis. Other potential applications could include pH-sensing and pH-triggered self-healing materials.

**Fig. 1:**
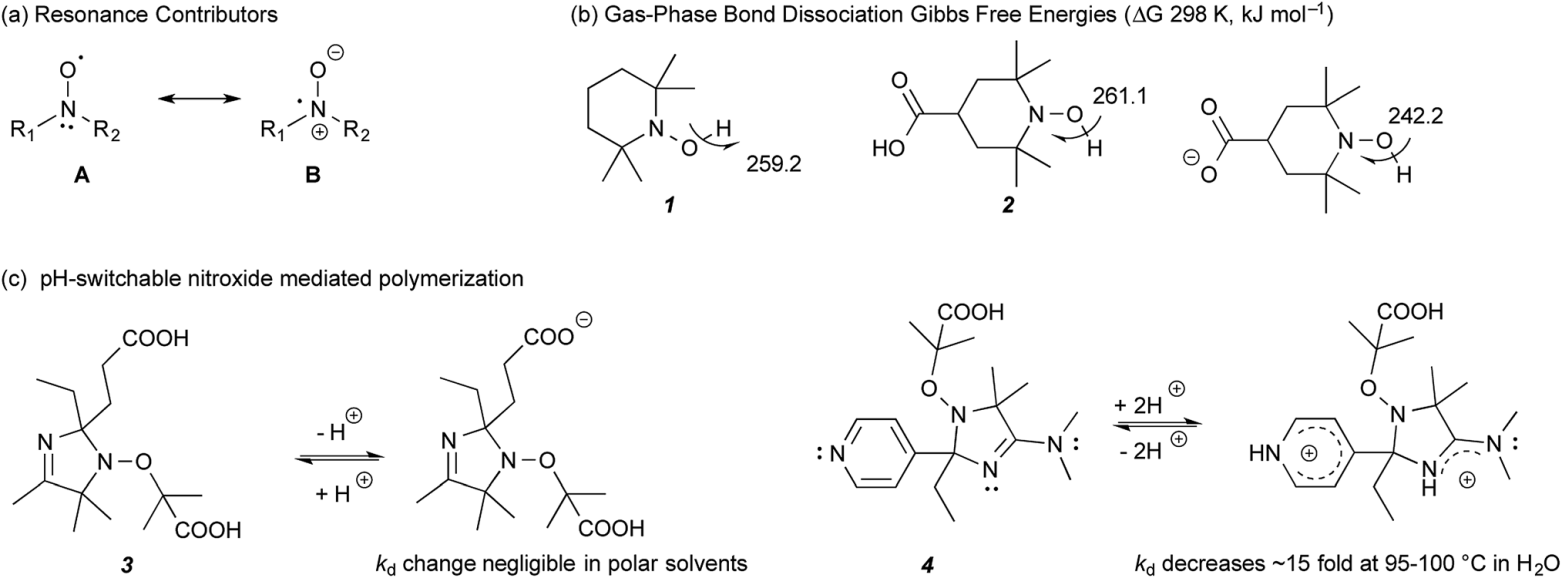
(a) Resonance contributors of a nitroxide radical. (b) Calculated bond dissociation Gibbs free energies (298 K, kJ mol^–1^) of non-substituted TEMPO-H (**1**) and protonated and deprotonated 4-CT-H (**2**) at 25 °C.^9^ (c) Literature studied pH-switchable nitroxides.^10^

Recently, we demonstrated that the stability of nitroxide radicals in the gas phase can be significantly enhanced by the presence of non-conjugated negatively charged groups in their vicinity; so much so, that the resulting radical anions also display SOMO–HOMO orbital conversion and oxidise preferentially to triplet diradicals instead of closed-shell zwitterions.^8^,^9^ The radical stabilising effect is electrostatic in origin and can be replicated, and even enhanced, if the negatively charged functional group is replaced with an equivalent point charge (*i.e.* an electric field). The enhanced stability arises because of the large degree of unpaired electron delocalisation between the nitrogen and oxygen atoms, resulting from resonance between the two contributors, A and B (Fig. 1a). Placing a negative charge on the ‘nitrogen side’ of the N–O bond stabilises contributor B, while replacing it with a positive charge, or placing it on the ‘oxygen side’ of N–O group instead, has a destabilising effect on B. Interaction of this dipole with a charge involves both a Coulombic (electrostatic) component and a resonance component, since stabilising or destabilising B impacts on the energy gap between A and B contributors and hence the amount of the resonance between them. When the negative charge is on the nitrogen side, the Coulombic and resonance effects reinforce each other, and result in the substantial radical stabilising effects, observed in these species. For example, the gas-phase room temperature bond dissociation Gibbs free energy for 4-carboxy-TEMPO (4-CT-H) decreases from 261.1 to 242.2 kJ mol^–1^ upon deprotonation of its carboxylic acid group, a so-called “pH switch” on radical stability of 18.9 kJ mol^–1^ (Fig. 1b).^8^,^9^

More generally, our calculations show that electrostatic effects on radical stability are broadly significant for several other families of delocalised radicals, even those lacking significant charge separated contributors to begin with. However, due to their electrostatic nature, their magnitude is expected to be strongly dependent on the polarity of the medium—with the largest effects expected in the gas phase, where the original experimental verification was carried out,^8^ and the smallest effects expected in highly polar solvents. Indeed our calculations predict that, while the gas-phase pH switches on radical stability should be largely (*ca.* 70–80%) preserved in a low polarity solvent such as toluene, the electrostatic pH switches are effectively quenched in water.^9^ In such cases significant pH switches on radical stability are still possible for specific compounds, but they are not strongly correlated with the gas-phase pH switches because they are dominated by more specific solute–solvent interactions.

Indeed, previous experimental studies have already discounted the presence of significant pH switches on nitroxide radical stability in polar solvents. For example, Edeleva *et al.*^10^ have shown that deprotonation of the carboxy groups on **3** had a negligible effect on the homolysis rate coefficient (*k*_d_) in water, though these same authors have demonstrated destabilising pH switches of up to a factor of 15 by protonating the two basic groups in **4**, which serves to destabilise resonance contributor B rather than stabilise it, as in the case of the negatively charged groups (Fig. 1c). More recently, Lucarini *et al.*,^11^ measured the equilibrium constant of H˙ exchange between 4-CT-H (**2**) and a reference alkoxyamine in the polar solvents acetonitrile and dimethyl sulfoxide and showed this was essentially independent of pH. Interestingly, analysis of the Van't Hoff's plots suggested that there was actually an appreciable enthalpic pH switch in acetonitrile, the less polar of the two solvents studied, but this was countered by an entropic contribution, which presumably arose through solvent–solute interactions rather than electrostatic effects. As these latter effects are likely to be system specific, the possibility of some degree of pH switching in acetonitrile for other alkoxyamines remains open, though it is clear that it is not large enough to overwhelm other, more conventional solvent effects.

To date all relevant experiments have confirmed the theoretically predicted large pH switches on the stability of carboxy-nitroxides in the gas-phase,^8^ as well as their disappearance in polar solvents.^11^ However, the critical question remains—can nitroxides be significantly stabilised by remote negatively charged functional groups in the relatively non-polar solvents, typically used in polymerisation and other synthetic settings? Or, more generally, are significant electrostatic effects on radical stability likely in other non-polar environments, such as enzyme active sites? In the present study we address this question and demonstrate significant and practically valuable pH-switches in *K*_eq_ on NO–H BDEs in the relatively low-polarity solvent dichloromethane (DCM). Moreover, our experiments allow us not only to quantify the pH switch on the thermodynamics of the H˙ atom transfer between 4-carboxy-TEMPO and a reference profluorescent nitroxide, but also demonstrate a significant pH switch on the kinetics of this reaction.

## Experimental methods

### Materials

The profluorescent nitroxide {2,2,6,6-tetramethyl-4-[(7-nitro-2,1,3-benzoxadiazol-4-yl)amino]-1-piperidinyl}oxidanyl radical (PFN-**5**),^12^ 1-hydroxy-2,2,6,6-tetramethylpiperidine-4-carboxylic acid (4-CT-H), 1-hydroxy-2,2,6,6-tetramethyl-4-piperidinone (TEMPONE-H) and 1-hydroxy-2,2,6,6-tetramethyl-4-[(7-nitro-2,1,3-benzoxadiazol-4-yl)amino]piperidine (PFN–**5**H) were synthesised according to the established methods as detailed in the ESI.† 12,13 Acetonitrile (ACN) and dichloromethane (DCM) were used as supplied from Merck. Triethylamine (TEA) was stored over 4 Å molecular sieves or potassium hydroxide prior to use.

### Time-dependent fluorescence spectroscopy

Three sets of reactions were studied: (a) the reaction of 4-CT-H (∼3.0 × 10^–5^ mM) and PFN-**5** (∼3.0 × 10^–5^ mM) in DCM; (b) the reaction of 4-CT-H (∼3.0 × 10^–5^ mM) and PFN-**5** (∼3.0 × 10^–5^ mM) in ACN; (c) the reaction of TEMPONE-H (∼3.0 × 10^–5^ mM) and PFN-**5** (∼3.0 × 10^–5^ mM) in DCM. For each set of reactions, stock solutions were prepared with the above concentrations, and the kinetics and thermodynamics of the hydrogen atom transfer reaction between the hydroxylamine (4-CT-H or TEMPONE-H) and PFN-**5** was studied in the absence of base, and with a series of increasing aliquots of base up to 200 equivalents, calculated relative to the starting hydroxylamine. For each experiment, the PFN-**5** solution (2 mL) was added to quartz fluorescence cuvettes and then either 4-CT-H solution (2 mL) or TEMPONE-H solution (2 mL) was added. Where relevant, TEA was added after the mixing of the two solutions and prior to the measurement of the fluorescence emission. The dilution effect of the TEA was taken into account when calculating reactant concentrations for the subsequent kinetic analysis.

The kinetics and thermodynamics of hydrogen atom transfer were studied by measuring the development of the fluorescence intensity of the product PFN-**5**H. Immediately upon mixing of the stock solutions, the cuvette was placed in a Cary Eclipse fluorescence spectrometer with temperature regulation carried out by a Cary Single Cell Peltier holder SPV1X0, and the emission fluorescence intensity was measured at 516 nm for the samples in DCM and 534 nm for the samples in ACN. In both cases the excitation wavelength was set to 350 nm. Measurements were made periodically over a period of up to three hours. The PFN-**5**H concentrations were determined from a calibration curve of PFN-**5**H emission intensity in the relevant solvent (DCM or ACN) at the respective temperature (25 or 10 °C) against concentration, and separate calibration curves were made for each concentration of base in the presence of 4-CT-H to account for the effect of TEA on the fluorescence of PFN-**5**H (details in the ESI†). The final (converged) concentration of PFN-**5**H from each sample was used to determine the equilibrium constant using eqn (1) and the kinetic data were fitted to a reversible second order kinetic model *via*eqn (2). A derivation of the kinetic model is provided in the ESI.†
1
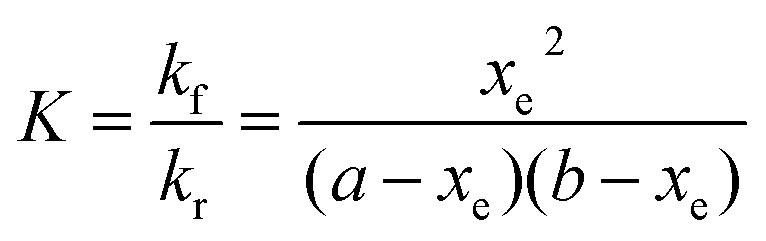

2
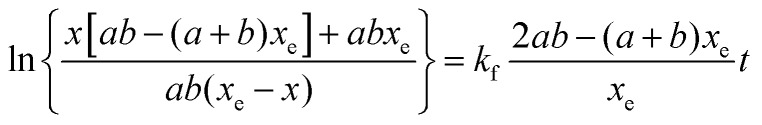
where: *a* = [PFN-**5**]_0_; *b* = [4-CT-H]_0_ or [TEMPONE-H]_0_; *x* = [PFN-**5**H] at any time *t*; and *x*_e_ = [PFN-**5**H] at equilibrium.

## Results and discussion

**Fig. 2:**
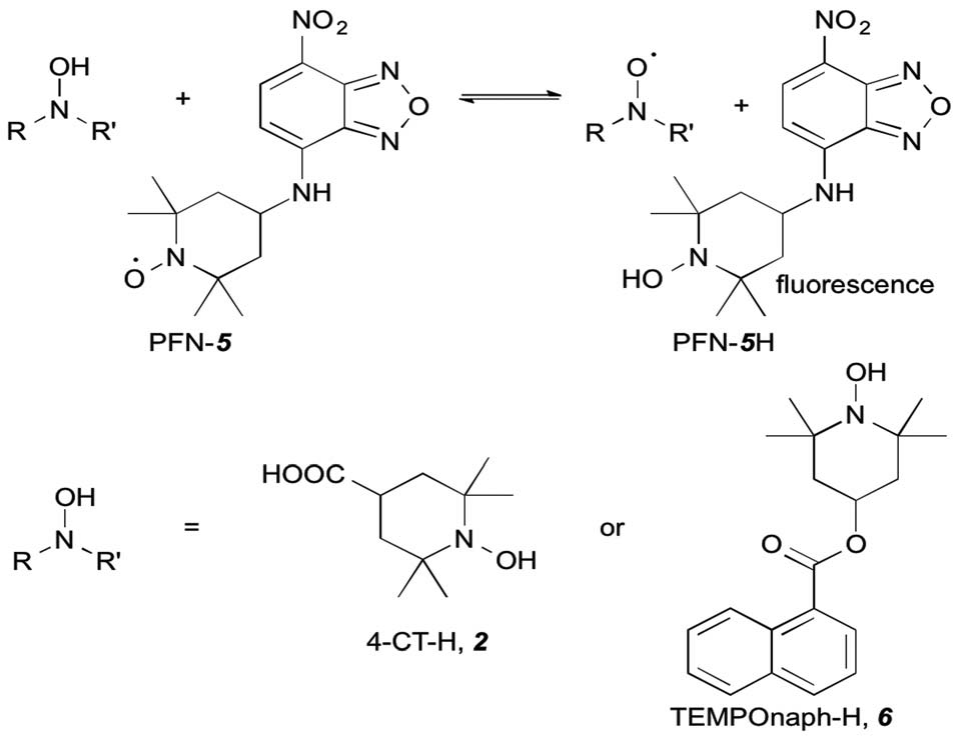
Hydroxylamine exchange of 4-CT-H or TEMPONE-H with the profluorescent nitroxide PFN-**5**.

In the work reported here we utilised the profluorescent nitroxide, {2,2,6,6-tetramethyl-4-[(7-nitro-2,1,3-benzoxadiazol-4-yl)amino]-1-piperidinyl}oxidanyl radical^12^ (PFN-**5**), to study the effect of pH on the O–H bond dissociation Gibbs free energy of 4-CT-H (Fig. 2). Profluorescent nitroxides (PFNs) are nitroxide radicals bearing a fluorophore functional group, the fluorescence of which is quenched by the unpaired electron; the signal is restored once the nitroxide traps another radical.^14^ As a result, the kinetics of the hydrogen atom transfer reaction between the 4-CT-H and PFN-**5** can be followed *via* the developing fluorescence signal as the non-fluorescent PFN-**5** is slowly converted into the fluorescent hydroxylamine PFN-**5**H. By performing the experiments with and without a base (triethylamine, TEA), we were able to ascertain whether or not deprotonation of the carboxylic acid group in 4-CT-H influences the hydrogen transfer reaction. To confirm that any pH effects on this reaction were due to the formation of a remote and negatively charged functional group, we also conducted a control reaction, in which 4-CT-H was replaced with an otherwise similar hydroxylamine that lacked an acid-base group, namely 1-hydroxy-2,2,6,6-tetramethyl-4-piperidinone (TEMPONE-H, **6**).

**Fig. 3:**
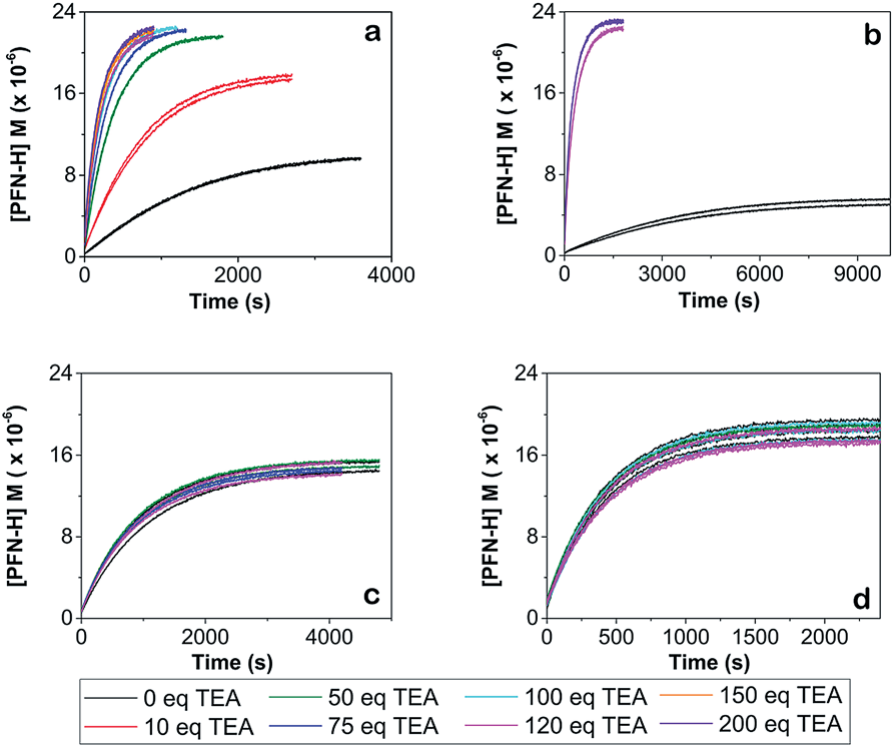
Concentration of PFN-**5**H *versus* time for the addition of triethylamine (TEA) at room temperature for (a) PFN-**5**/4-CT-H system in dichloromethane at 25 °C, (b) the same system at 10 °C, (c) PFN-**5**/4-CT-H in acetonitrile at 25 °C, and (d) the PFN-**5**/TEMPONE-H control in dichloromethane at 25 °C. In the PFN-**5**/4-CT-H system in dichloromethane at either temperature (graphs a and b) increasing concentrations of base up to 120 eq. dramatically increases the rate of reaction and shifts the equilibrium to the right. Beyond 120 eq. additional base has no effect and the curves become indistinguishable. In the polar solvent (graph c) or the control (graph d) the presence or absence of the base and its concentration has no significant effect on the kinetics and thermodynamics and all curves are indistinguishable.

**Fig. 4:**
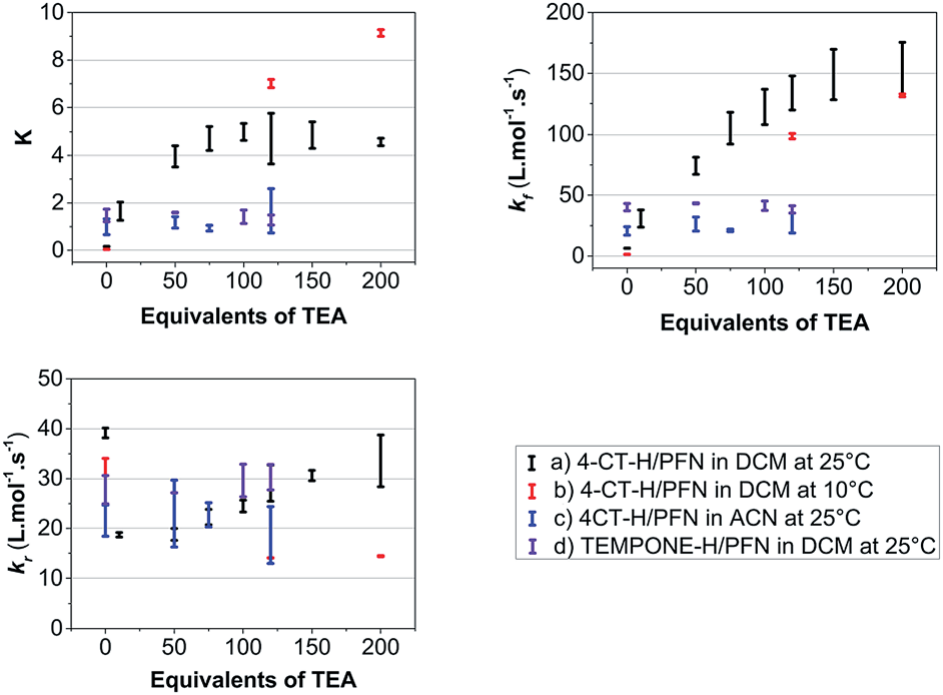
Average values of the equilibrium constants (*K*) and forward (*k*_f_) and reverse (*k*_r_) rate constants as a function of the equivalents of triethylamine (TEA) base added for (a) the PFN-**5**/4-CT-H reaction in dichloromethane at 25 °C, (b) the same reaction at 10 °C, (c) the same reaction in acetonitrile at 25 °C, and (d) for the PFN-**5**/TEMPONE-H control reaction in dichloromethane at 25 °C. The error bars are the calculated standard deviations of replicate samples.

Fig. 3 shows the concentration of the fluorescent PFN-**5**H as a function of time in the presence of different amounts of the TEA base for the reaction with 4-CT-H in dichloromethane, and TEMPONE-H in dichloromethane. Dichloromethane is a relatively non-polar solvent (dielectric constant *ε* = 8.9), while acetonitrile is relatively polar (*ε* = 37.5). The time dependent concentrations of PFN-**5**H were determined from the measured fluorescence intensity in conjunction with separate calibration experiments (see ESI†). Calibration curves were constructed for each effective concentration of base used so as to ensure that the results were not an artefact of any pH effects on the fluorescence intensity of the PFN-**5**H product. For each experiment, the final equilibrium concentrations of PFN-**5**H were used to estimate the equilibrium constant, while the time dependent concentrations were fitted with a reversible second order kinetic model to determine the forward and reverse rate coefficients. The resulting kinetic and thermodynamic parameters are plotted as a function of the equivalents of base in Fig. 4. Tables of raw data and full details of the experiments and analysis are provided in the ESI.†

It is clear that in the reaction of 4-CT-H with the PFN-**5** in dichloromethane, the addition of base increases both the rate at which equilibrium is achieved and the final equilibrium concentrations of PFN-**5**H. These effects depend on the amount of base added up to approximately 120 equivalents, at which point the carboxylic acid is presumably fully deprotonated.

The pH switches can be quantified by comparing the average values of each parameter at 120 equivalents of base to the corresponding values under otherwise identical conditions in the absence of base (Table 1). For the 4-CT-H reaction in dichloromethane at 25 °C, we find that the equilibrium constant shifts to the right by a factor of 28.7 upon full deprotonation of the remote carboxy group of 4-CT-H, corresponding to an increase in stability of the resulting distonic radical anion by approximately one order of magnitude. Interestingly, at 10 °C this pH switch increases by a further order of magnitude to 168.2. This temperature dependence, also noted in the recent work of Lucarini *et al.*,^11^ implies a significant opposing entropic contribution to the pH switch, which presumably arises from more specific solute–solvent interactions.

**Table 1:** Calculated equilibrium constants (*K*) (dimensionless) and forward (*k*_f_) and reverse (*k*_r_) rate constants (L mol^–1^ s^–1^) as a function of the equivalents of triethylamine (TEA) base added for (a) the PFN-**5**/4-CT-H reaction in dichloromethane (DCM) at 25 °C, (b) the same reaction at 10 °C, (c) the same reaction in acetonitrile (MeCN) at 25 °C, and (d) for the PFN-**5**/TEMPONE-H control reaction in DCM at 25 °C. The pH switch is calculated as the ratio of corresponding values at 120 equivalents of base and with no base present

	*K*			*k*_f_/L mol^–1^ s^–1^			*k*_r_/L mol^–1^ s^–1^		
	No base	120 eq.	pH switch	No base	120 eq.	pH switch	No base	120 eq.	pH switch
(a) DCM 25 °C	0.16 ± 0.01	4.70 ± 1.06	28.7 ± 6.5	6.4 ± 0.2	133.8 ± 14.0	20.9 ± 2.2	39.1 ± 1.0	29.1 ± 3.6	0.7 ± 0.1
(b) DCM 10 °C	0.04 ± 0.01	7.01 ± 0.17	168.2 ± 20.6	1.3 ± 0.1	98.7 ± 2.2	73.6 ± 5.2	32.3 ± 1.7	14.1 ± 0.1	0.4 ± 0.1
(c) MeCN 25 °C	1.00 ± 0.33	1.67 ± 0.93	1.68 ± 1.09	20.7 ± 3.5	27.2 ± 8.1	1.3 ± 0.5	21.5 ± 3.1	18.7 ± 5.7	0.9 ± 0.3
(d) Control 25 °C	1.47 ± 0.26	1.28 ± 0.21	0.87 ± 0.21	40.2 ± 3.0	38.4.2 ± 3.0	1.0 ± 0.1	27.8 ± 2.8	30.3 ± 2.5	1.1 ± 0.1

At both temperatures, the significant pH switch on the reaction thermodynamics is largely preserved in the reaction kinetics, implying that the transition state stabilisation is similar in magnitude to that of the product radical. Whilst the unpaired electron density is distributed further from the negative charge in the transition state, this is presumably countered to a large extent by its greater polarizability. More generally, the greater polarizability of transition states, compared with stable species, implies that electrostatic catalysis of radical reactions could be significant even when the reactant and product radicals are not themselves especially delocalised provided there are appropriately placed charges in the vicinity of the reaction centre. Since the transition state and product radical are stabilised to similar extents, the pH switch on the reverse reaction is consequently relatively small at both temperatures.

In contrast to the above-mentioned results, the addition of base had little or no effect on the kinetics and thermodynamics of the hydrogen transfer reaction between TEMPONE-H and the PFN-**5** under the same conditions, confirming that the pH effects in 4-CT-H are associated with deprotonation of its acid-base group rather than, for example, base catalysis of the hydrogen transfer reaction *via* a sequential proton loss electron transfer (SPLET) mechanism^15^ or some other degradation of the reagents by the added base. Consistent with the recent experimental work of Lucarini *et al.*,^11^ the pH switches in acetonitrile are also essentially negligible. This further confirms the electrostatic origin of the pH effects on the 4-CT nitroxide radical stability, which are predicted *via* theoretical calculations^9^ to be progressively quenched in increasingly polar environments.

## Conclusion

In conclusion, we have demonstrated experimentally significant (up to 2 orders of magnitude) pH switching of radical stability in the common organic solvent dichloromethane. That is, despite the presence of solvent, deprotonation of a non-conjugated carboxylic acid that is approximately 6 Å away^9^ from the formal radical centre significantly increases the stability of the radical relative to the corresponding closed shell hydroxylamine. Using a simple thermodynamic cycle^8^ it can be shown that these results also imply that the acidity of the remote carboxylic acid group is increased by as much as 2 p*K*_a_ units in the nitroxide radical *versus* the hydroxyloxyamine under these conditions. Moreover, in this work we have also demonstrated equally significant electrostatic catalysis by a remote negatively charged functional group on the hydrogen transfer reaction between two locally equivalent nitroxide radicals.

More generally, these findings validate our recent theoretical predictions^9^ that electrostatic stabilisation of delocalised radicals remains practically significant in low polarity environments, such as those relevant to synthesis and polymerisation processes. These stabilisation effects are not limited to nitroxide radicals but are present to smaller or greater extents in all delocalised radicals, including those derived from phenols, lipids, peptides and nucleic bases.^9^ Hence, demonstrating that these effects persist in solution raises the possibility of a broad range of synthetic applications in which the protonation state of non-conjugated acid-base groups on a substrate or auxiliary is used to influence the kinetics and/or thermodynamics of a radical reaction.

Although the stabilisation effects demonstrated here are practically useful, they are nonetheless significantly attenuated when compared with those previously demonstrated in the gas phase,^8^ and with those predicted computationally in yet lower polarity solvents such as toluene.^9^ In the present work, the use of lower polarity solvents was precluded by the limited solubility of the deprotonated 4-CT. Instead, dichloromethane was chosen as it represented a successful compromise between solubility on the one hand and a sizeable electrostatic effect on the other. We anticipate that these electrostatic effects could by further optimized in this and other systems by placing the charged groups closer to the reaction centre. In this way, the stabilising effects (which decay rapidly with distance)^9^ are larger to begin with, the screening effect of the solvent becomes less significant, and the use of more polar solvents is then possible. However, it is worth noting that this compromise is avoided altogether in enzymes, which are able to bind reagents within active sites that can be both low in polarity, and have substantial local electric fields created by charged residues.^16^,^17^ Mimicking this approach to electrostatic catalysis, using heterogeneous catalysts, or avoiding negatively charged functional groups altogether and using, instead, external electric fields may be necessary to harness the full potential of electrostatic catalysis in synthetic chemistry.

## Supplementary Material

Supplementary information

## References

[cit1] Hawker C. J., Bosman A. W., Harth E. (2001). Chem. Rev..

[cit2] Gryn'ova G., Ingold K. U., Coote M. L. (2012). J. Am. Chem. Soc..

[cit3] Bobbitt J. M., Brückner C., Merbouh N. (2010). Org. React..

[cit4] Soule B. P., Hyodo F., Matsumoto K., Simone N. L., Cook J. A., Krishna M. C., Mitchell J. B. (2007). Free Radical Biol. Med..

[cit5] Gryn'ova G., Barakat J. M., Blinco J. P., Bottle S. E., Coote M. L. (2012). Chem.–Eur. J..

[cit6] Suga T., Konishi H., Nishide H. (2007). Chem. Commun..

[cit7] Azarkh M., Singh V., Okle O., Seemann I. T., Dietrich D. R., Hartig J. S., Drescher M. (2013). Nat. Protoc..

[cit8] Gryn'ova G., Marshall D. L., Blanksby S. J., Coote M. L. (2013). Nat. Chem..

[cit9] Gryn'ova G., Coote M. L. (2013). J. Am. Chem. Soc..

[cit10] Edeleva M. V., Kirilyuk I. A., Zhurko I. F., Parkhomenko D. A., Tsentalovich Y. P., Bagryanskaya E. G. (2011). J. Org. Chem..

[cit11] Franchi P., Mezzina E., Lucarini M. (2014). J. Am. Chem. Soc..

[cit12] Becker M., De Cola L., Studer A. (2011). Chem. Commun..

[cit13] Jones M. J., Moad G., Rizzardo E., Solomon D. H. (1989). J. Org. Chem..

[cit14] Blinco J. P., Fairfull-Smith K. E., Morrow B. J., Bottle S. E. (2011). Aust. J. Chem..

[cit15] Litwinienko G., Ingold K. U. (2007). Acc. Chem. Res..

[cit16] Warshel A., Sharma P. K., Kato M., Xiang Y., Liu H., Olsson M. H. M. (2006). Chem. Rev..

[cit17] Fried S. D., Bagchi S., Boxer S. G. (2014). Science.

