# Supplementary material for: Experimental demonstration of pH-dependent electrostatic catalysis of radical reactions
Source: Chem Sci. 2015 Jun 23;6(10):5623–7. doi: 10.1039/c5sc01307k (PMC5949849; doi:10.1039/c5sc01307k)
Supplement: Supplementary file 1 [file SC-006-C5SC01307K-s001.pdf]

Electronic Supplementary Material (ESI) for Chemical Science.
This journal is © The Royal Society of Chemistry 2015

Supporting Information

Experimental Demonstration of pH-Dependent Electrostatic Catalysis of Radical Reactions

Marta Klinska,[1] Leesa M. Smith,[1] Ganna Gryn'ova,[1] Martin G. Banwell[1] and Michelle L. Coote[1,2]*

[1]*Research School of Chemistry, Institute of Advanced Studies, Australian National University, Canberra ACT 2601, Australia*

[2]*ARC Centre of Excellence for Electromaterials Science*

Email: michelle.coote@anu.edu.au

Table of Contents

Appendix S1. Experimental Procedures 2
Appendix S2 Fluorescence Calibration Curves 5
Appendix S3. Kinetic Analysis 8
Appendix S4 Kinetic Model Fitting Plots 9
Appendix S5. Kinetic and Thermodynamic Data 20
  Table S1. Exchange Reaction of PFN-*5* and 4-CT-H in dichloromethane at 25°C 20
  Table S2. Exchange Reaction of PFN-*5* and 4-CT-H in dichloromethane at 10°C 22
  Table S3. Exchange Reaction of PFN-*5* and 4-CT-H in acetonitrile at 25°C 23
  Table S4. Exchange reaction of PFN-*5* and TEMPONE-H in dichloromethane at 25°C 24
Appendix S6. NMR Spectra of Synthesised Compounds 25
Appendix S7. References 31

Appendix S1. Experimental Procedures

Materials. The profluorescent nitroxide, 2,2,6,6-tetramethyl-4-([7-nitro-2,1,3-benzoxadiazol-4-yl)amino]-1-piperidinyloxy radical (PFN-*5*), 1-hydroxy-2,2,6,6-tetramethylpiperidine-4-carboxylic acid (4-CT-H), 1-hydroxy-2,2,6,6-tetramethyl-4-piperidinone (TEMPONE-H) and 1-hydroxy-2,2,6,6-tetramethyl-4-[(7-nitro-2,1,3-benzoxadiazol-4-yl)amino])piperidine (PFN-*5*H) were synthesised according to the methods described below. Acetonitrile was supplied by Merck and used as received. Dichloromethane was supplied by Merck and dried over activated 4 Å molecular sieves prior to use. Triethylamine (TEA) was stored over 4 Å molecular sieves and potassium hydroxide prior to use.

General methods. NMR spectra were recorded at 298 K using a Bruker AVANCE 400 spectrometer. The resonance due to residual chloroform (δ 7.26 ppm) or residual dimethyl sulfoxide (δ 2.50 ppm) was used as the internal reference for ^{1}H NMR spectra recorded in these solvents. Coupling constants (*J*) are quoted to the nearest 0.1 Hz. The central signal of the multiplet arising from the solvent $CDCl_3$ (δ 77.1 ppm) or $(CD_3)_2SO$ (δ 39.5 ppm) was used as the internal reference for ^{13}C NMR spectra recorded in these solvents. When necessary, the assignment of proton signals was assisted by HSQC experiments while the assignment of carbon signals was, in some instances, assisted by APT, HSQC and/or HMBC experiments. IR spectra (apart from that of TEMPONE-H) were recorded on a Perkin-Elmer Spectrum One spectrometer as neat films on NaCl plates for oils or as KBr disks for solid products. The FT-IR spectra of TEMPONE-H was recorded on a Perkin-Elmer Spectrum One spectrometer as a dry powder. Mass spectra were recorded by the staff within the Mass Spectrometry Unit of the Research School of Chemistry, Australian National University, Canberra using a VG Autospec M series sector (EBE) spectrometer operating in low resolution EI mode, a VG Quattro II triple quadrupole machine operating in low resolution ESI mode and/or a Bruker Apex3 4.7T FTICR-MS operating in high resolution ESI mode. Microanalyses were performed within the Microanalytical Laboratory, Research School of Chemistry, Australian National University, Canberra. Melting points were measured on Optimelt automated melting point apparatus and are uncorrected. Analytical TLC was performed using 20 x 70 mm silica gel 60 F_{254} (0.2 μm) coated aluminium sheets that had been cut from larger ones supplied by Merck (A.T. 5554). Flash chromatography employed Merck Kiesegel 60 (230–400 mesh) silica gel. Reactions were conducted under a positive pressure of dry argon or nitrogen in oven-dried glassware. Diethyl ether, toluene and THF were dried over sodium wire and distilled from sodium benzophenone ketyl. Dichloromethane was distilled from calcium hydride. Commercially available chemicals were purified by standard procedures or used as purchased.

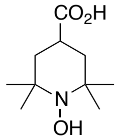

1-Hydroxy-2,2,6,6-tetramethylpiperidine-4-carboxylic acid: A magnetically stirred solution of 4-carboxy-TEMPO (200 mg, 1 mmol) in dry MeOH (5 mL) was treated, in one portion, with *L*-ascorbic acid (176 mg, 1 mmol, 1.0 mole eq.). The reaction mixture was stirred at room temperature for 5 min. then concentrated under reduced pressure and the residue was taken up in CH_2Cl_2 (10 mL) and the resulting solution was washed with water (1 x 10 mL) and brine (1 x 10 mL) then dried (Na_2SO_4), filtered and concentrated under reduced pressure to give the title compound (156 mg, 78%) as off-white solid, mp = 195 °C. ^{1}H NMR [400 MHz, $(CD_3)_2SO$] δ 1.02 (s, 6H), 1.06 (s, 6H), 1.37-1.45 (complex m, 2H), 1.69-1.72 (complex m, 2H), 2.50-2.57 (complex m, 1H), 3.27 (broad s, 1H), 7.18 (s, 1H); ^{13}C NMR [101 MHz, $(CD_3)_2SO$] δ 19.2 (4 x CH_3), 32.4 (CH_2), 34.0 (CH_2), 41.6 (CH), 57.3 (2 x C), 176.4 (C); IR (KBr disc) ν_{max} 3600-2400 (broad), 3435, 2985, 2951, 1404 cm^{-1}; MS (ESI+) *m/z* (%): 202 (100, $[M+H]^+$); HRMS (ESI+) *m/z* calculated for $C_{10}H_{19}NO_3$ $[M+H]^+$:202.1443, found: 202.1443.

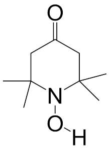

1-Hydroxy-4-Oxo-2,2,6,6-tetramethyl-1-piperidine: A magnetically stirred solution of 4-oxo-TEMPO (229 mg, 1.35 mmol) in MeOH (5 mL) was treated, in one portion, with *L*-ascorbic acid (257 mg, 1.46 mmol, 1.08 mole eq.). The reaction mixture was stirred at room temperature for 15 min. and then concentrated under reduced pressure. The residue was taken up in water (10 mL) and the resulting solution was extracted with diethyl ether (3 x 5 mL) and then dried ($MgSO_4$), filtered and concentrated under reduced pressure. The crude product was then recrystallised from n-hexane to give the title compound (63.9mg, 28%) mp = 86-88 °C. ^{1}H NMR (400 MHx, CDCl3) δ 1.19, 12H), 2.39 (s, 4H), 4.60 (broad s, 1H); ^{13}C NMR (101 MHz, CDCl3) δ 25.9 (4 x CH_3), 52.80 (2 x CH_2), 61.4 (2 x C), 207.4 (C=O); IR (powder) ν_{max} 3389, 3330, 2972, 2937, 1729, 1702, 1466, 1419, 1375, 1362, 1301, 1251, 1233, 1224 cm^{-1}, MS (ESI+) *m/z* (%): 172 [100, $(M+H)^+$]; HRMS (ESI+) *m/z* calculated for $C_9H_{18}NO_2$ $[M+H]^+$: 172.1338 found: 172.1337

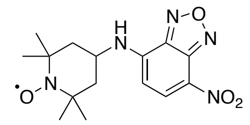

2,2,6,6-Tetramethyl-4-([7-nitrobenzo[*c*][1,2,5]oxadiazol-4-yl]-amino)piperidin-1-oxyl radical: A magnetically stirred solution of 4-chloro-7-nitrobenzofurazan (200 mg, 1 mmol) in dry ethyl acetate (2 mL) was treated, dropwise, with triethylamine (139 μL, 1 mmol, 1.0 eq) then 4-amino-TEMPO (171 mg, 1 mmol, 1.0 eq). The resulting reaction mixture was stirred under a nitrogen atmosphere for 3 h then diluted with ethyl acetate (10 mL). The resulting solution was washed with water (1 x 10 mL) and brine (1 x 10 mL) before being dried (Na_2SO_4), filtered and concentrated under reduced pressure. The resulting light-yellow oil was subjected to flash column chromatography (silica, 1:9 v/v ethyl acetate/hexane elution) to give the title compound[2] (164 mg, 49%) as a bright-orange powder, m.p. = 227 °C. IR (KBr disc) ν_{max} 3214, 3063, 2978, 1578, 1530, 1492, 1317, 1282, 1261, 1237, 1185, 1027 cm^{-1}; MS (ESI+) m/z (%): 357 [100, $[M+Na]^+$], MS (ESI-) m/z (%): 334 [100, $(M-H)^-$]; HRMS (ESI+) *m/z* calculated for $C_{15}H_{20}N_5O_4^{\bullet}$ $[M+Na]^+$: 357.1413 found: 357.1413; Elemental analysis: $C_{15}H_{20}N_5O_4^{\bullet}$ calculated: C, 52.88; H, 6.03; N, 20.95; found: C, 53.92; H, 6.19; N, 20.94.

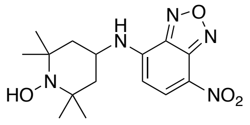

1-hydroxy-2,2,6,6-tetramethyl-4-[(7-nitro-2,1,3-benzoxadiazol-4-yl)amino])piperidine: A magnetically stirred solution of 1-oxyl-2,2,6,6-tetramethyl-4-((7-nitrobenzo[*c*][1,2,5]oxadiazol-4-yl)amino)piperidine radical (50 mg, 0.150 mmol, 1.0 eq) in dry MeOH (2 mL) was treated, in one portion, with *L*-ascorbic acid (28 mg, 0.157 mmol, 1.05 mole eq.). The ensuing mixture was stirred for 30 min at room temperature then concentrated under reduced pressure to give a red oil. A solution of this material in dichloromethane (2 mL) was washed with water (1 x 2 ml) and brine (1 x 2 mL) before being dried (Na_2SO_4), filtered and concentrated under reduced pressure. The resulting red oil

was subjected to flash column chromatography (silica, 1:5 → 1:1 ethyl acetate/dichloromethane gradient elution) to give the title compound (39 mg, 78%) as a red powder, m.p. 218-222 °C: ^{1}H NMR (400 MHz, $(CD_3)_2SO$) δ 1.10 (s, 6H), 1.17 (s, 6H), 1.68-1.87 (complex m, 4H), 4.06 (broad s , 1H), 6.45 (s, 1H), 7.23 (s, 1H), 8.48 (broad s, 1H), 9.26 (broad s, 1H); ^{13}C NMR (101 MHz, $(CD_3)_2SO$) δ 19.5 (2 x CH_3), 32.5 (2 x CH_3), 43.3 (2 x CH_2), 45.4 (CH), 57.8 (2 x C), 99.2 (CH), 120.6 (C), 138.0 (CH), 144.1 (C), 144.4 (C), (one signal due to a quaternary carbon obscured or overlapping); IR (KBr disc) ν_{max} 3500-3300 (broad), 3346, 2977, 2930, 1620, 1568, 1499, 1320, 1286, 1269 cm^{-1}; MS (ESI+) *m/z* (%): 336 [100, $(M+H)^+$], 358 (80, [$(M+Na)^+$], MS (ESI-) *m/z* (%): 333 [100, $(M-2H)^{2-}$]; HRMS (ESI+) *m/z* calculated for $C_{15}H_{21}N_5O_4$ $[M+H]^+$: 336.1672 found: 336.1671, $[M+Na]^+$: 357.1413 found: 357.1414; Elemental analysis: $C_{15}H_{21}N_5O_4$ calculated: C, 53.72; H, 6.31; N, 20.88; found: C, 53.48; H, 6.66; N, 20.29.

Determination of equilibrium constant and rate constants in dichloromethane for reaction between 4-CT-H and PFN-*5*. Solutions of 4-carboxy-TEMPO-H (~6.0 x 10^{-5} M) and profluorescent nitroxide (PFN-*5*) (~6.0 x 10^{-5} M) were made up in dichloromethane. The PFN-*5* solution (2 mL) was added to quartz fluorescence cuvettes and then 4-carboxy-TEMPO-H solution (2 mL) was added to this solution. The cuvette was then placed in a Cary Eclipse fluorescence spectrometer with temperature controlled by a Cary Single Cell Peltier Holder SPV 1 x 0 and the emission fluorescence intensity was measured at 516 nm (the excitation wavelength was set to 350 nm). Measurements were made periodically over a period of up to 3 h. For the samples in which triethylamine was added, this was added after the mixing of the two solutions and prior to the measurement of the fluorescence emission. The number of equivalents of base is given relative to the concentration of 4-carboxy-TEMPO-H in solution. All concentrations were determined from a calibration curve of PFN-*5*H emission intensity in dichloromethane against concentration and separate calibration curves were made for each concentration of base in the presence of 4-CT-H to account for the effect of TEA on the fluorescence of PFN-*5*H. The final concentration of PFN-*5*H from each sample was used to determine the equilibrium rate constant and the kinetic data was fitted to a reversible second order kinetic model. All samples were corrected for the dilution effect of the addition of TEA.

Determination of equilibrium constant and rate constants in acetonitrile for reaction between 4-CT-H and PFN-*5*. Solutions of 4-carboxy-TEMPO-H (~6.0 x 10^{-5} M) and profluorescent nitroxide (PFN-5-5) (~6.0 x 10^{-5} M) were made up in acetonitrile. PFN-*5* solution (2 mL) was added to quartz fluorescence cuvettes and then 4-carboxy-TEMPO-H solution (2 mL) was added to this solution. The cuvette was then placed in a Cary Eclipse fluorescence spectrometer with temperature controlled by a Cary Single Cell Peltier Holder SPV 1 x 0. The excitation wavelength was set at 350 nm and the emission fluorescence intensity was measured at 534 nm. Measurements were made periodically over a period of up to 2 h. For the samples in which TEA was added, this was added after the mixing of the two solutions and prior to the measurement of the fluorescence emission. The number of equivalents of base is given relative to the concentration of 4-carboxy-TEMPO-H in solution. All concentrations were determined from a calibration curve of PFN-*5*H emission intensity in acetonitrile in the presence of 4-CT-H against concentration and separate calibration curves were constructed for each concentration of TEA. The final concentration of PFN-*5*H from each sample was used to determine the equilibrium rate constant and the kinetic data was fitted to a reversible second order kinetic model (see below). All samples were corrected for the dilution effect of the addition of TEA.

Determination of equilibrium constant and rate constants in dichloromethane for reaction between TEMPONE-H and PFN-*5*. Solutions of TEMPONE-H (~6.0 x 10^{-5} M) and profluorescent nitroxide (PFN-5-5) (~6.0 x 10^{-5} M) were made up in dichloromethane. PFN-*5* solution (2 mL) was added to quartz fluorescence cuvettes and then 4-carboxy-TEMPO-H solution (2 mL) was added to

this solution. The cuvette was then placed in a Cary Eclipse fluorescence spectrometer with the temperature controlled by a Cary Single Cell Peltier Holder SPV 1 x 0. The sample excitation wavelength was set at 350 nm. The emission fluorescence intensity was measured at 516 nm and samples were taken periodically over a period of up to 1 h. For the samples in which TEA was added, this was added after the mixing of the two solutions and prior to the measurement of the fluorescence emission. The number of equivalents of base is given relative to the concentration of 4-carboxy-TEMPO-H in solution. All concentrations were determined from a calibration curve of PFN-**5**H emission intensity in dichloromethane against concentration and separate calibration curves were made for each concentration of base in the presence of TEMPONE-H to account for the effect of triethylamine (TEA) on the fluorescence of PFN-**5**H. The final concentration of PFN-**5**H from each sample was used to determine the equilibrium rate constant of the second order equilibrium and the kinetic data was fitted to a reversible second order kinetic model with equilibrium (see below). All samples were corrected for the dilution effect of the addition of TEA.

Appendix S2 Fluorescence Calibration Curves

Calibration of the response of intensity of the maximum fluorescence of PFN-**5**H in all solvent solutions were done in the presence of 0.03 mM 4-carboxy-TEMPO-H. This concentration was chosen as this is the identical concentration of 4-carboxy-TEMPO-H during the exchange experiments. The intensity of the emission from the PFN-**5**H fluorophore was measured with subsequent additions of a 10% solution of TEA in solvent to allow for the reduction in fluorescence with increasing pH in solution.

PFN-**5**H with 0.03 mM of 4-CT-H in dichloromethane at 25°C

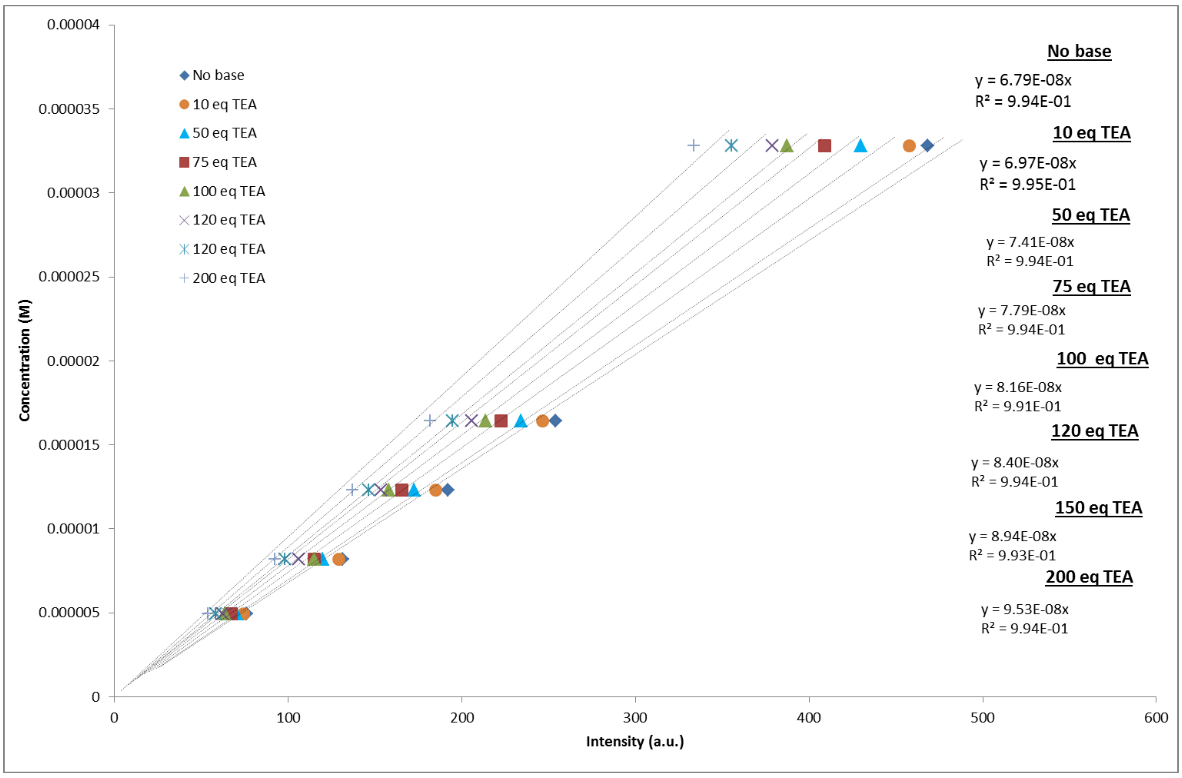

PFN-**5**H with 0.03 mM of 4-CT-H in dichloromethane at 10°C

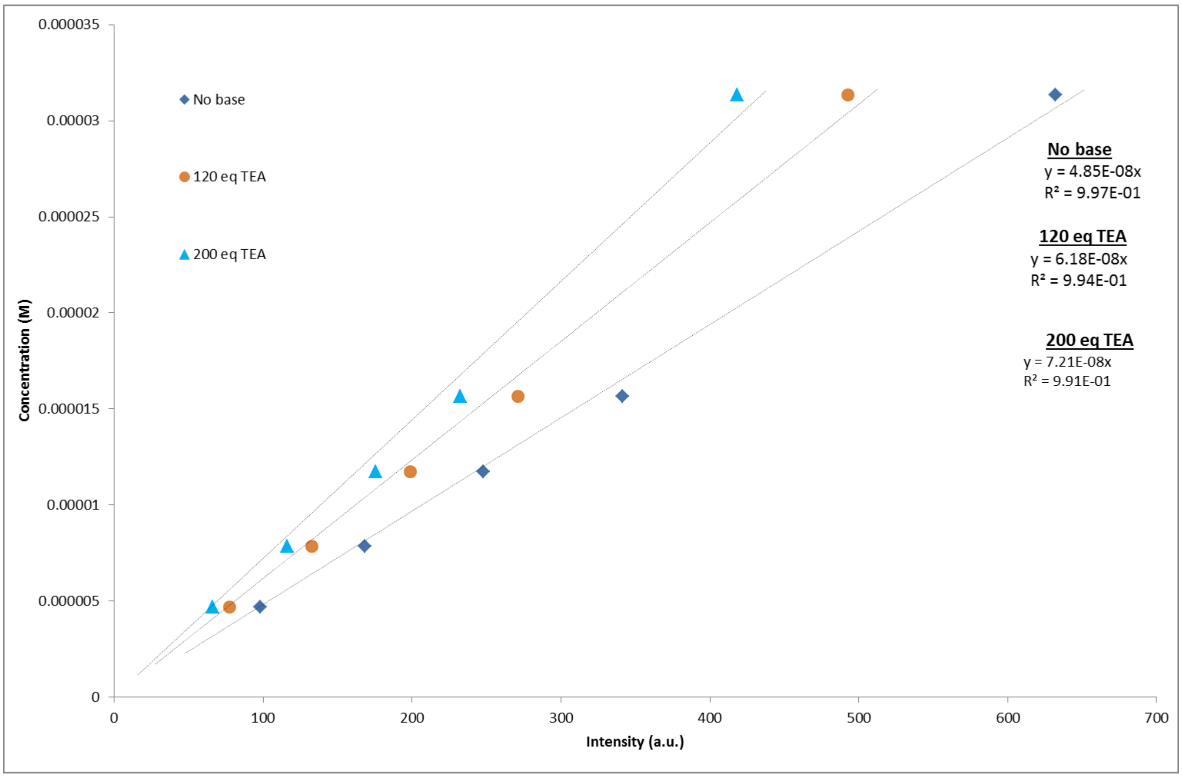

PFN-**5**H with 0.03 mM of 4-CT-H in acetonitrile at 25°C

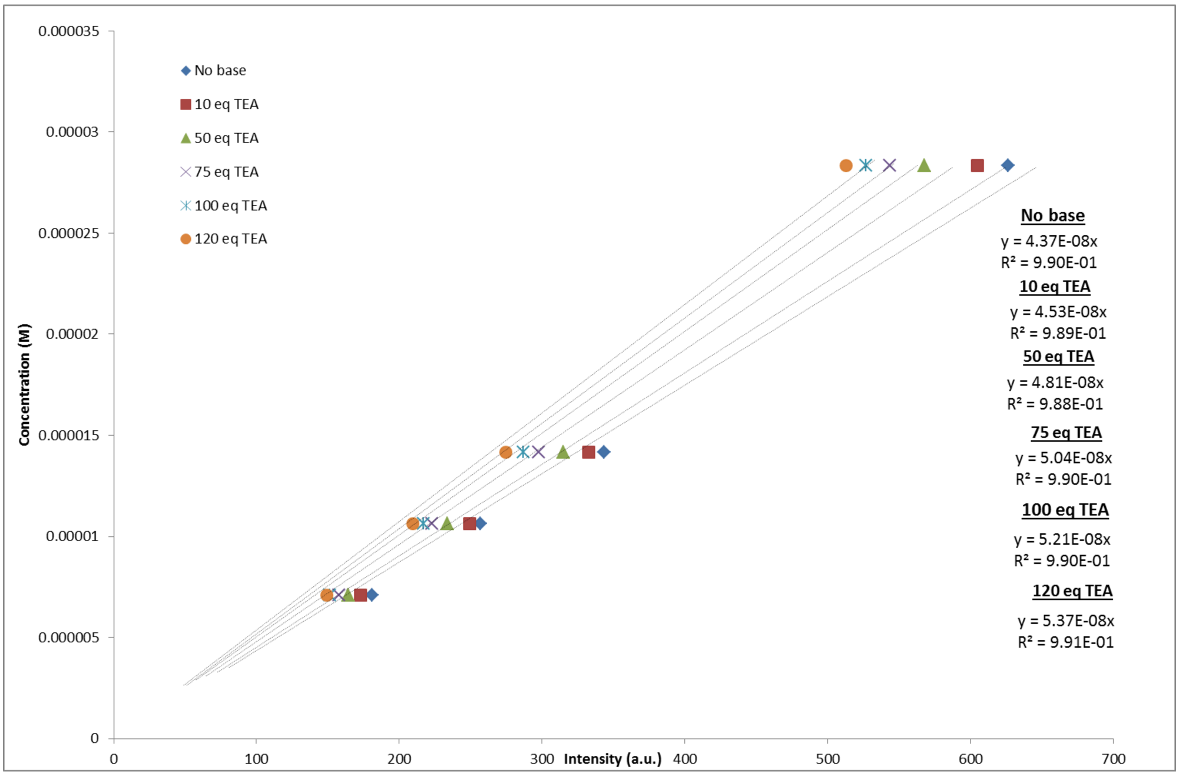

PFN-**5**H with 0.03 mM of TEMPONE-H in dichloromethane at 25°C

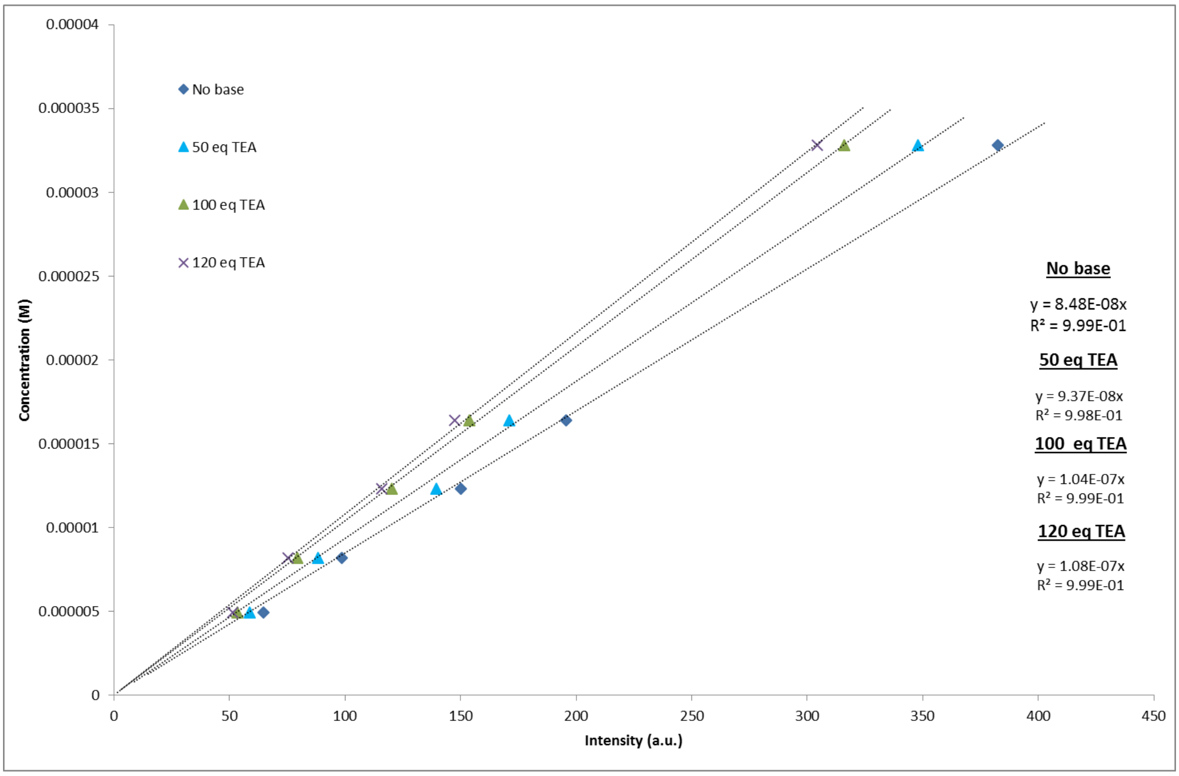

Appendix S3. Kinetic Analysis

For the reversible hydrogen atom transfer reaction (1), the rate at which the fluorescent PFN-*5*H is produced is given by equation (2).

$$\mathrm{PFN} - \mathbf{5} + 4 - \mathrm{CT} - \mathrm{H} \underset{k_r}{\overset{k_f}{\rightleftarrows}} \mathrm{PFN} - \mathbf{5}\mathrm{H} + 4 - \mathrm{CT} \quad (1)$$

$$\frac{d\,[\mathrm{PFN{-}5H}]}{dt} = k_f[\mathrm{PFN} - \mathbf{5}][4 - \mathrm{CT} - \mathrm{H}] - k_r[\mathrm{PFN} - \mathbf{5}\mathrm{H}][4 - \mathrm{CT}] \; (2)$$

If, as in the present work, only PFN-*5* and 4-CT-H are present at the beginning of the reaction, then expressing this reaction in terms of the extent of reaction, x, gives equation (3).

$$\frac{dx}{dt} = k_f[([\mathrm{PFN} - \mathbf{5}]_o - x)([4 - \mathrm{CT} - \mathrm{H}]_0 - x)] - k_r x^2 \qquad (3)$$

By definition, at equilibrium;

$$K = \frac{k_f}{k_r} = \frac{x_e^2}{([\mathrm{PFN{-}5}]_0 - x_e)([4{-}\mathrm{CT}{-}\mathrm{H}]_0 - x_e)} \qquad (4)$$

Substituting for k_r into equation (3) gives

$$\frac{dx}{dt} = k_f[([\mathrm{PFN} - \mathbf{5}]_o - x)([4 - \mathrm{CT} - \mathrm{H}]_0 - x)] - k_f \frac{([\mathrm{PFN{-}5}]_0 - x_e)([4{-}\mathrm{CT}{-}\mathrm{H}]_0 - x_e)}{x_e^2} x^2 \qquad (5)$$

Letting, $[\mathrm{PFN{-}}\mathit{5}]_0 = \mathrm{a}$ and $[\mathrm{4{-}CT{-}H}]_0 = \mathrm{b}$, this can then be integrated by partial fractions to give

$$ln\left\{\frac{x[ab-(a+b)x_e]+ab\,x_e}{ab(x_e-x)}\right\} = k_f \frac{2ab-(a+b)x_e}{x_e} t \qquad (6)$$

If $[\mathrm{PFN}]_0 = [\mathrm{4{-}CT{-}H}]_0 = \mathrm{a}$; then equation (6) simplifies to

$$ln\left\{\frac{x[a-2x_e]+a\,x_e}{a(x_e-x)}\right\} = k_f \frac{2a(a+b)x_e}{x_e} t \qquad (7)$$

Adapted From John W. Moore, Ralph G. Pearson, Kinetics and Mechanism, 3rd Edition, John Wiley & Sons, 1981 ISBN 0-471-03558-0

Appendix S4 Kinetic Model Fitting Plots

PFN-5/4-CT-H system in dichloromethane at 25°C

Plots of $ln\left\{\frac{x[ab-(a+b)x_e]+ab\ x_e}{ab(x_e-x)}\right\}$ versus time, slope = $k_f\left(\frac{2ab-(a+b)x_e}{x_e}\right)$

No base (0 eq TEA)

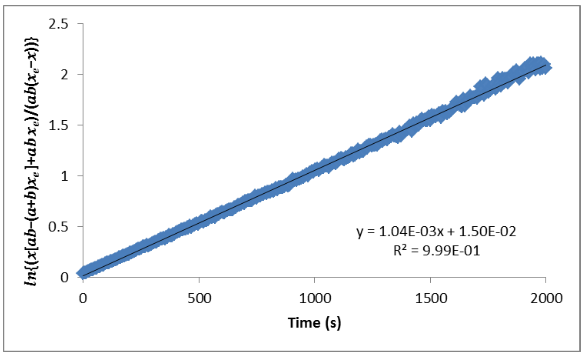

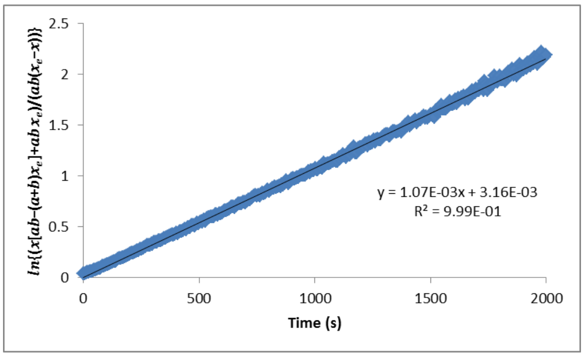

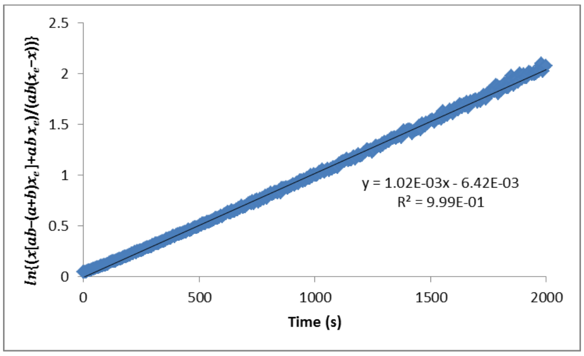

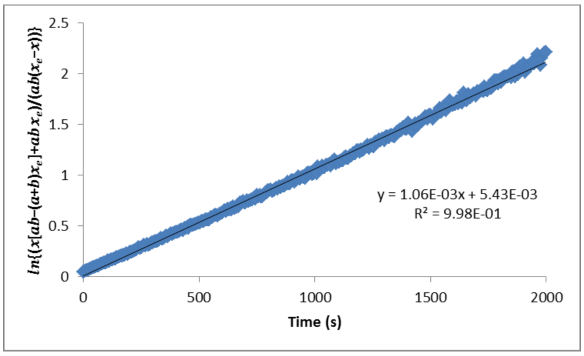

10 eq TEA

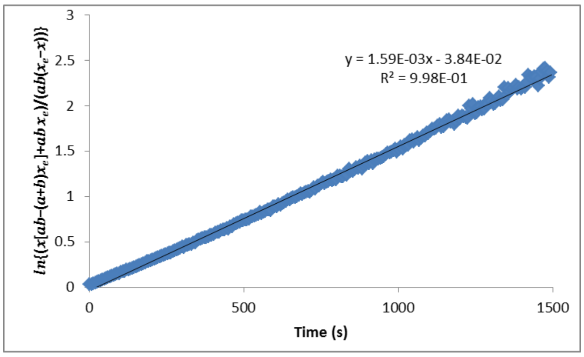

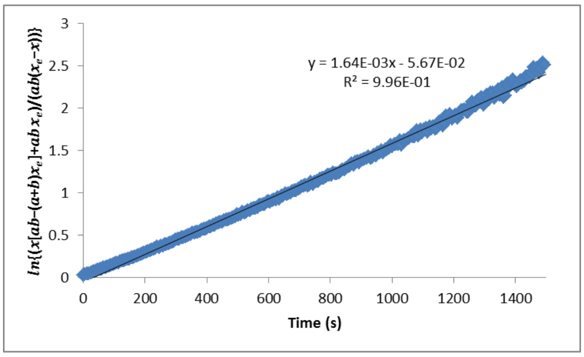

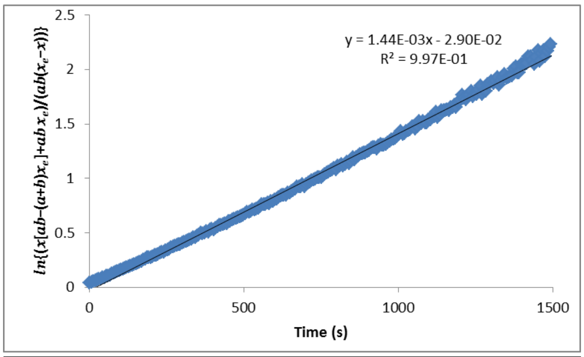

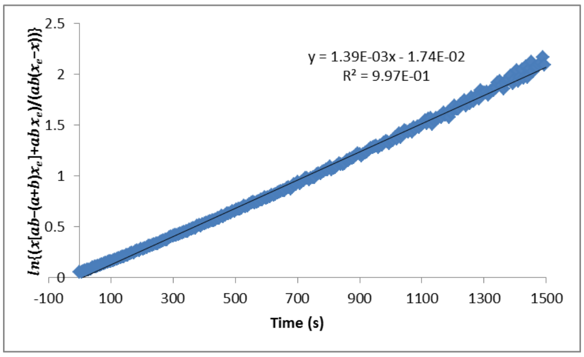

50 eq TEA

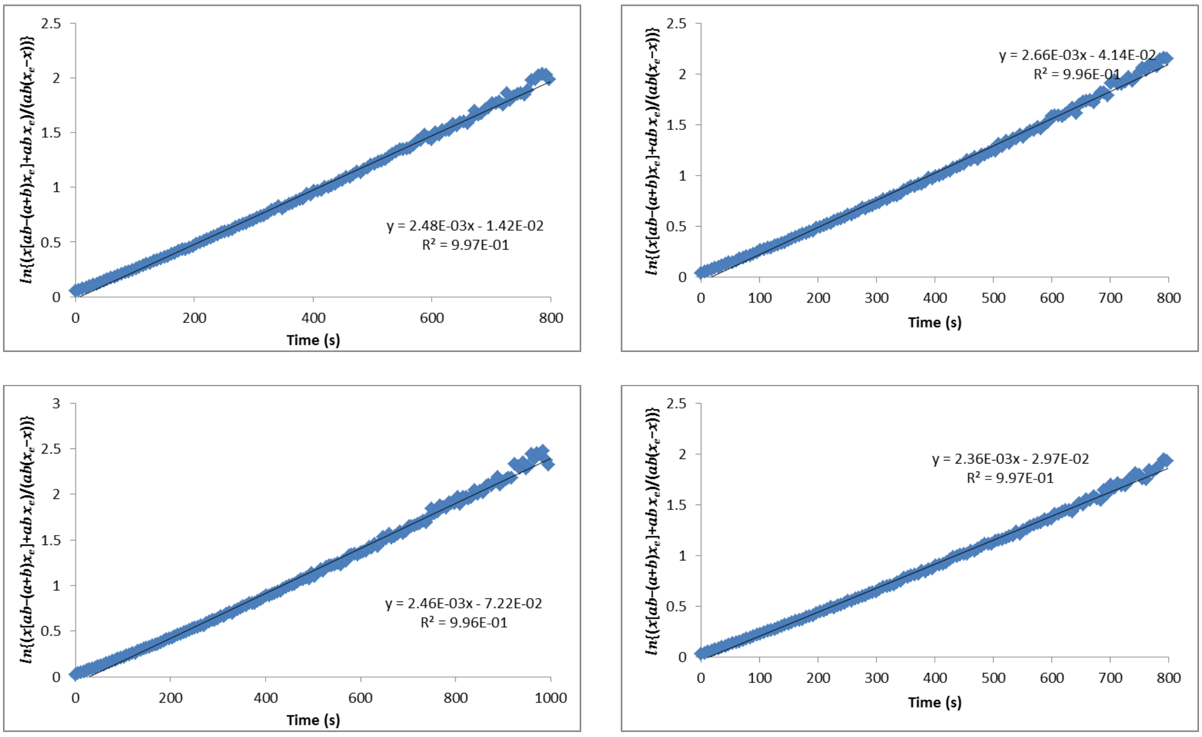

75 eq TEA

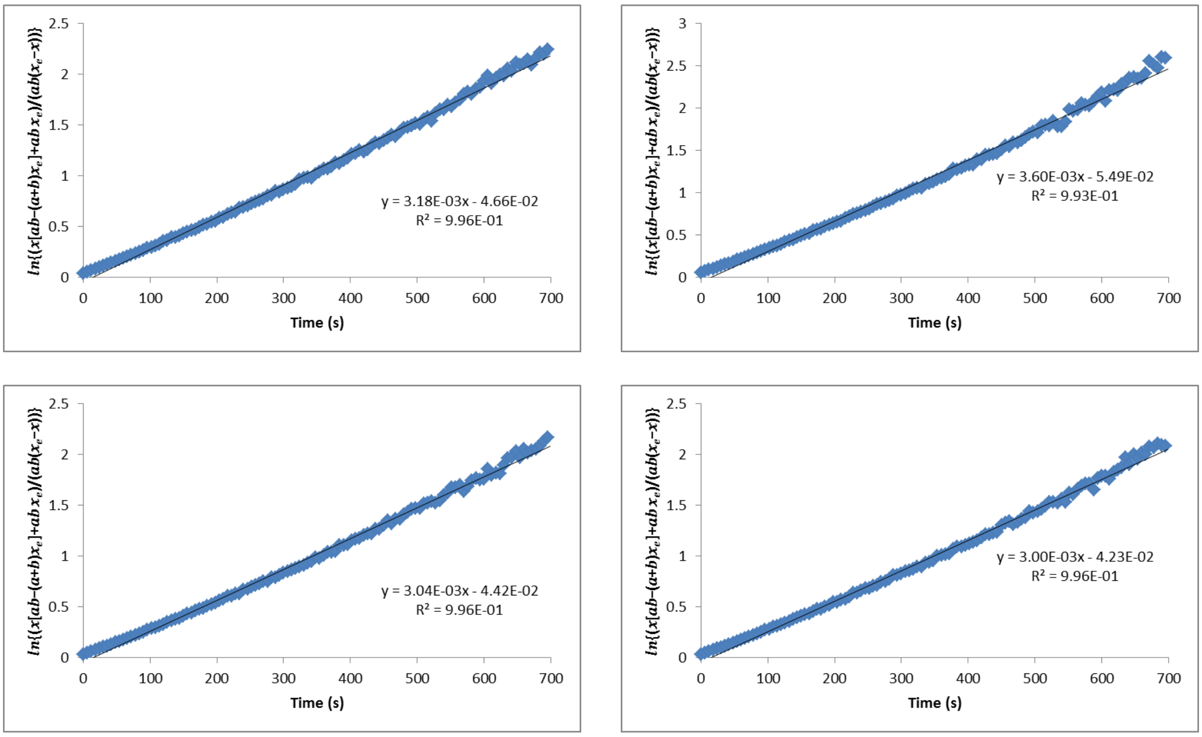

100 eq TEA

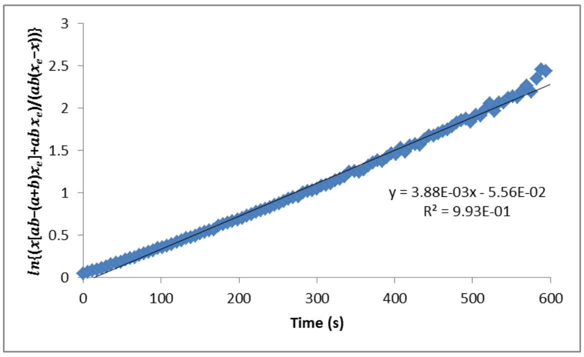

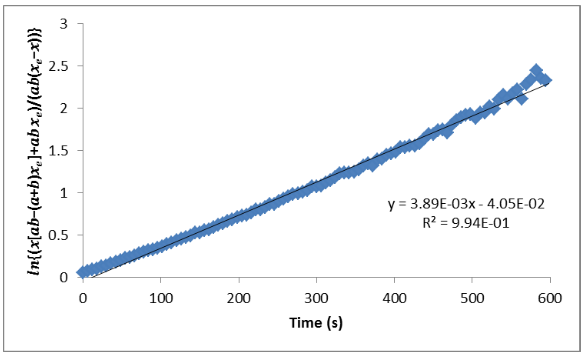

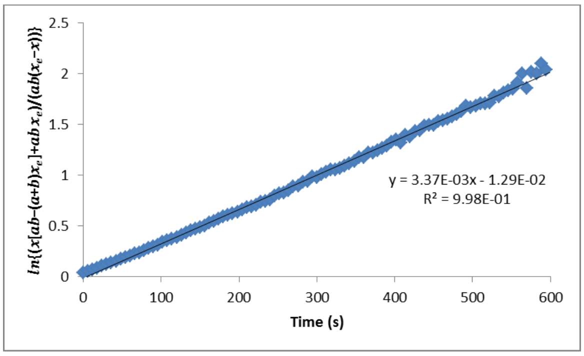

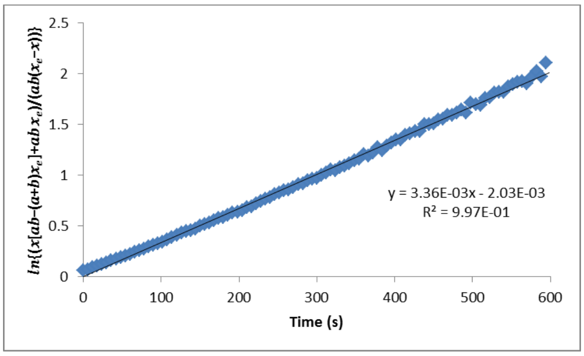

120 eq TEA

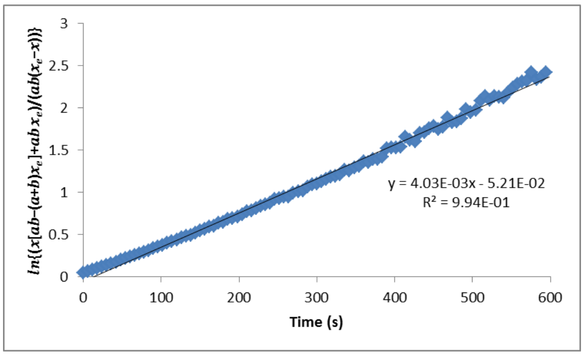

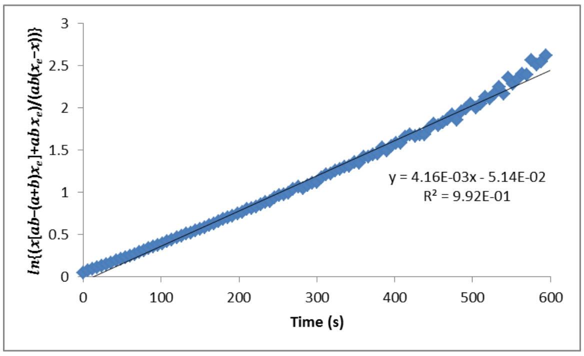

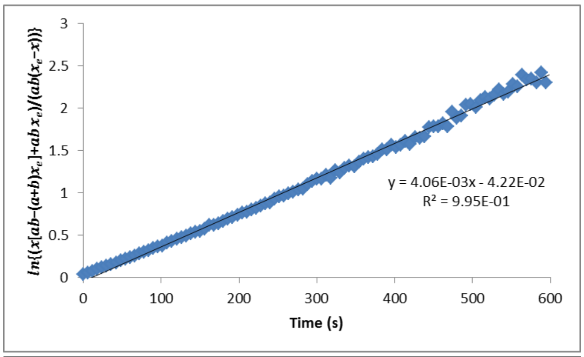

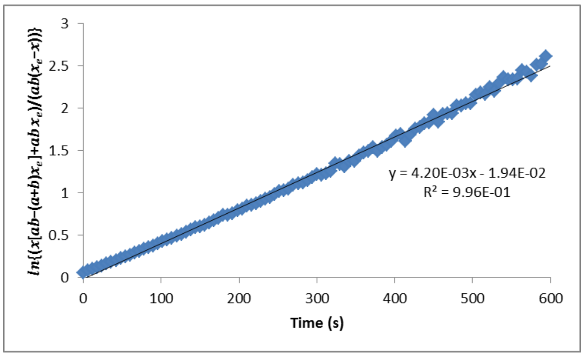

150 eq TEA

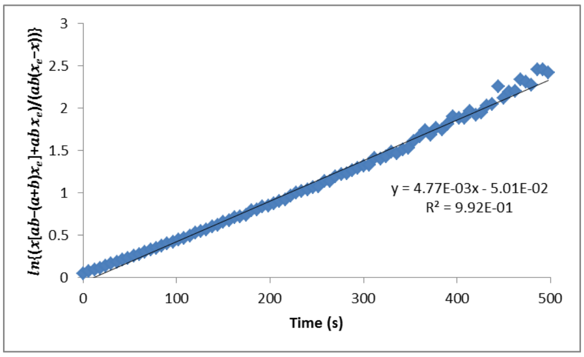

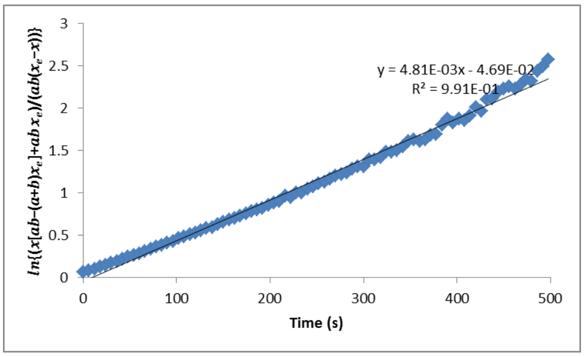

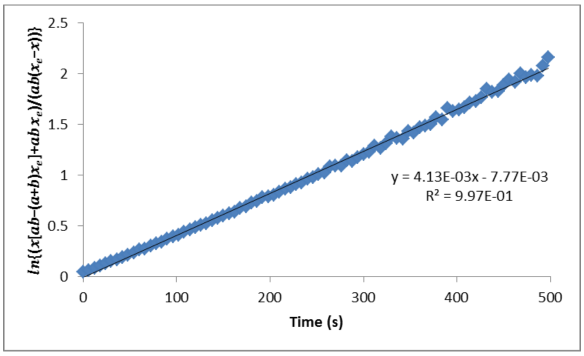

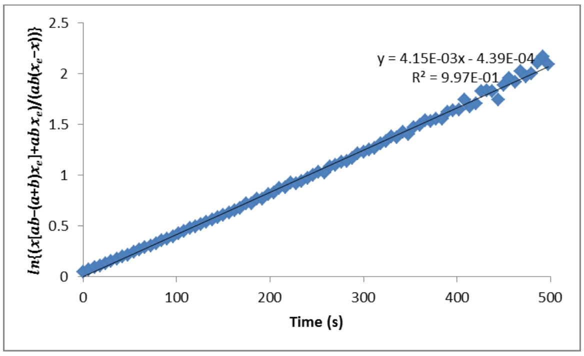

200 eq TEA

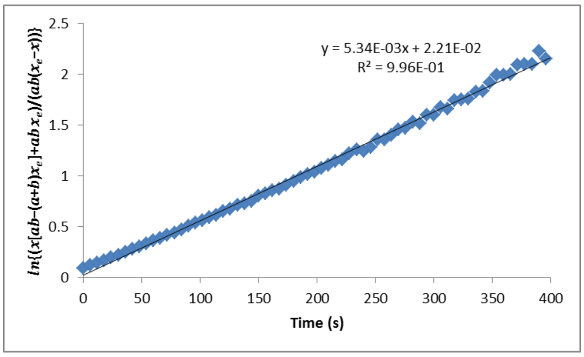

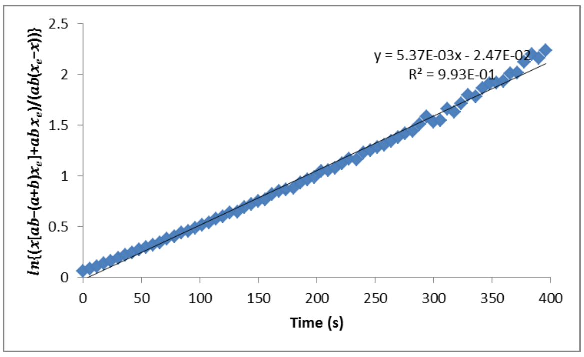

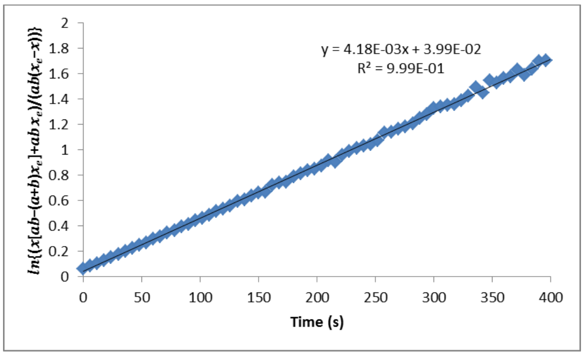

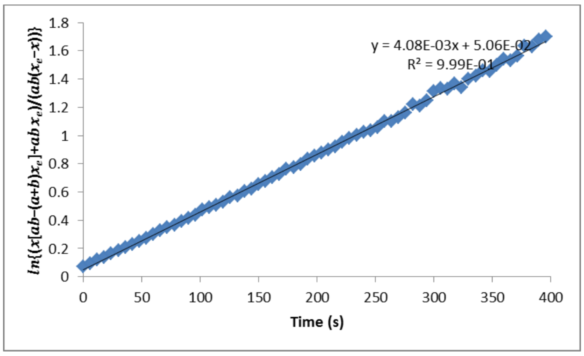

PFN-5/4-CT-H system in dichloromethane at 10° C

Plots of $ln\left\{\frac{x[ab-(a+b)x_e]+ab\ x_e}{ab(x_e-x)}\right\}$ versus time, slope = $k_f\left(\frac{2ab-(a+b)x_e}{x_e}\right)$

No base (0 eq TEA)

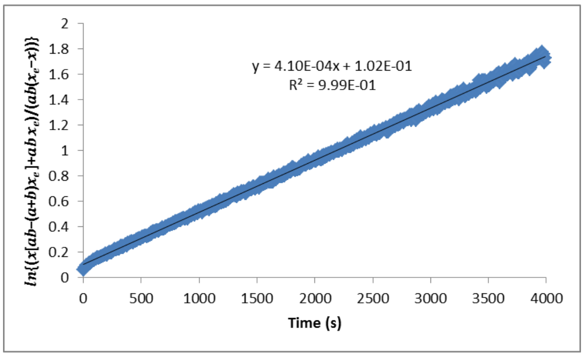

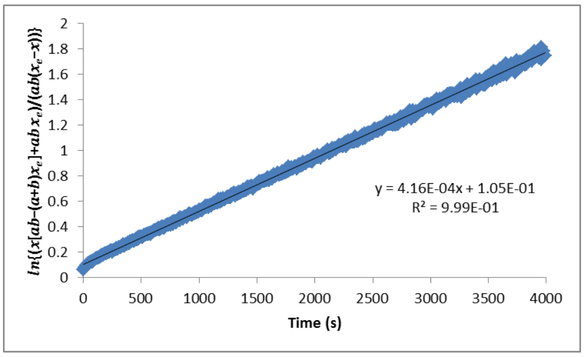

120 eq TEA

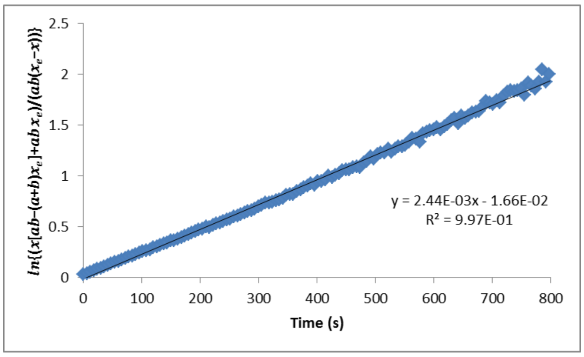

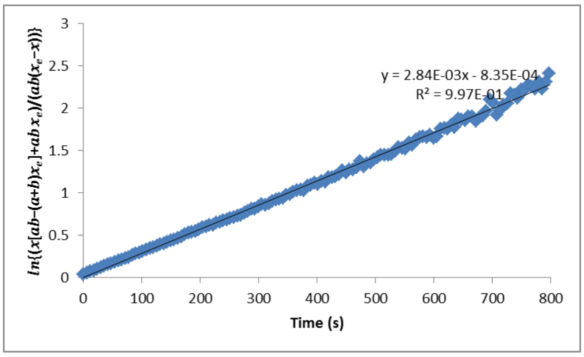

PFN-5/4-CT-H system in acetonitrile at 25°C

Plots of $ln\left\{\frac{x[ab-(a+b)x_e]+ab\,x_e}{ab(x_e-x)}\right\}$ versus time; slope = $k_f\left(\frac{2ab-(a+b)x_e}{x_e}\right)$

No base (0 eq TEA)

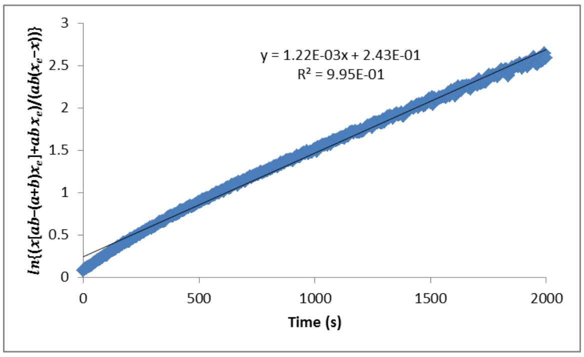

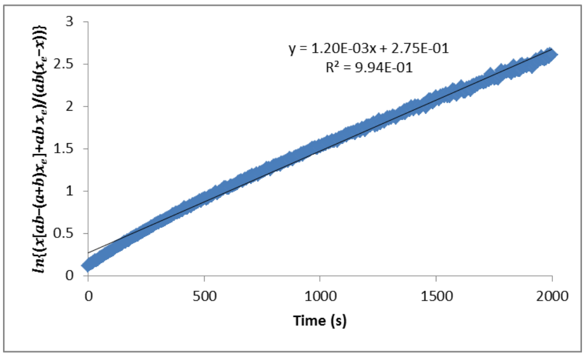

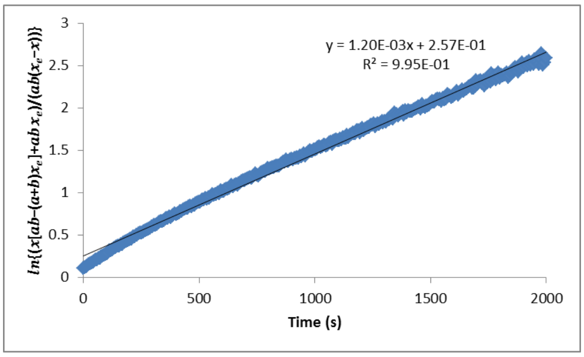

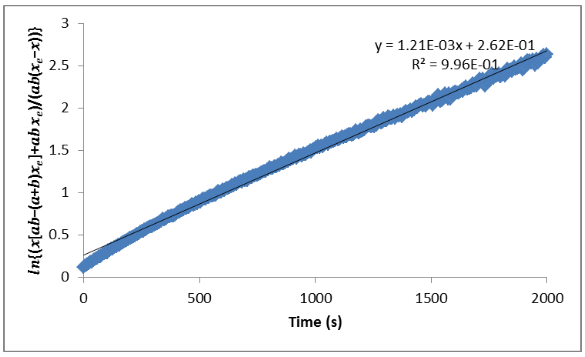

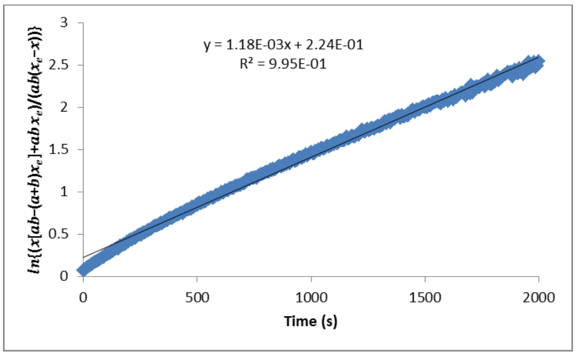

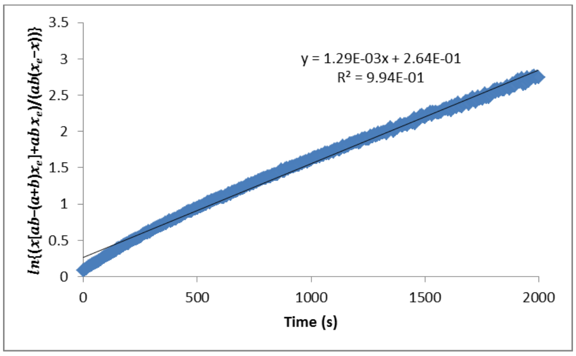

50 eq TEA

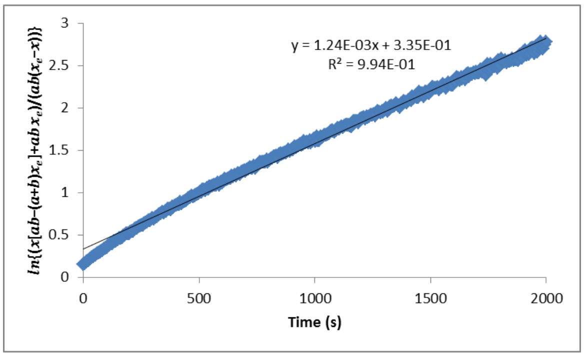

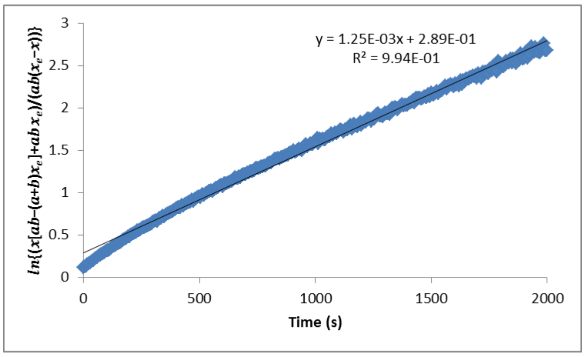

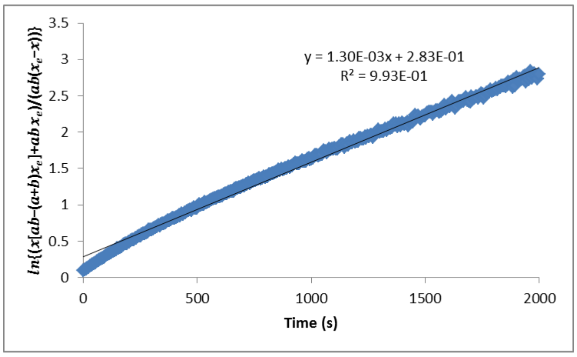

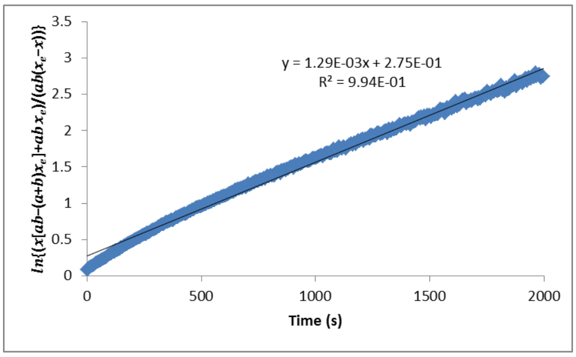

75 eq TEA

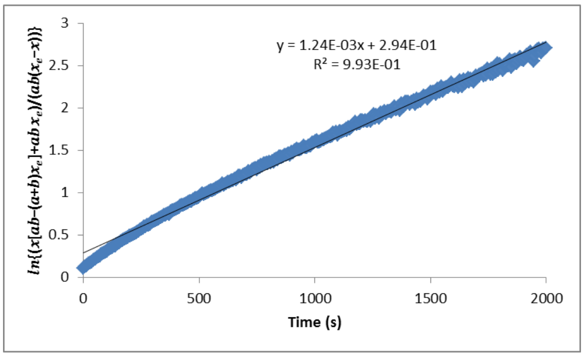

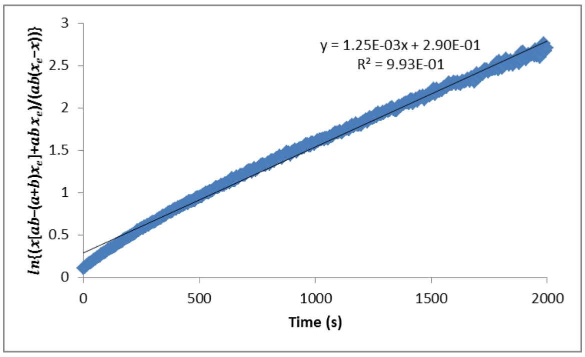

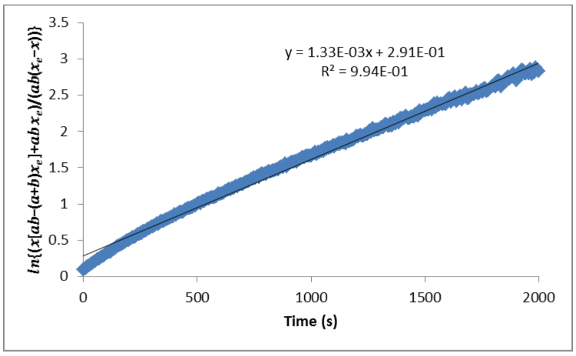

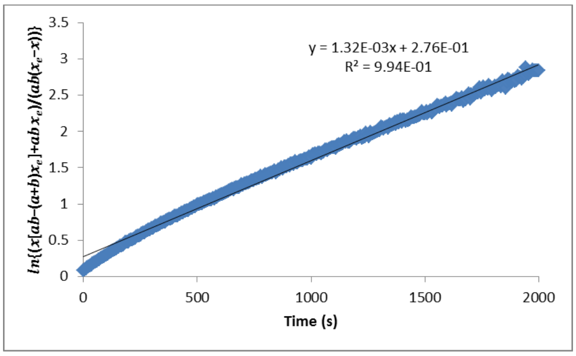

120 eq TEA

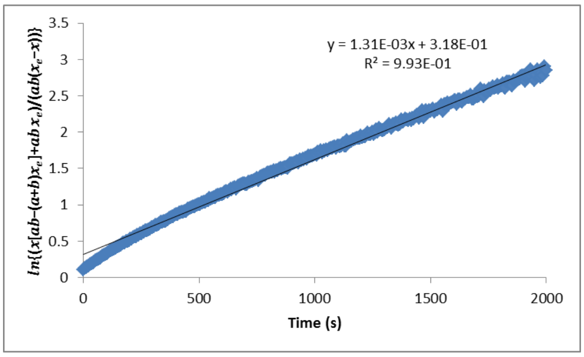

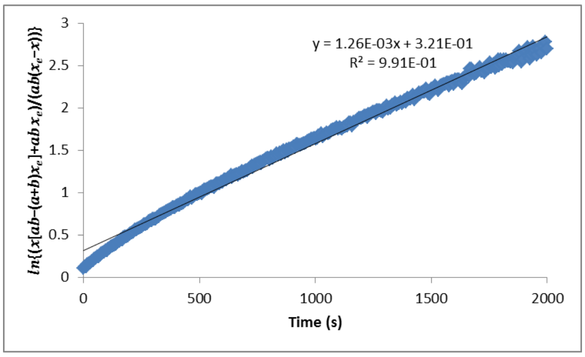

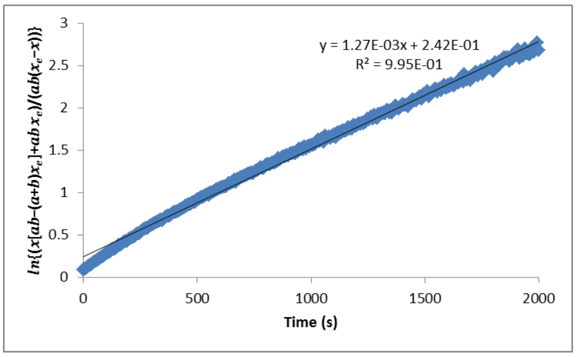
y = 1.27E-03x + 2.42E-01
R² = 9.95E-01
ln{(x[ab−(a+b)xe]+ab xe)/(ab(xe−x))}
Time (s)
0
0.5
1
1.5
2
2.5
3
0
500
1000
1500
2000

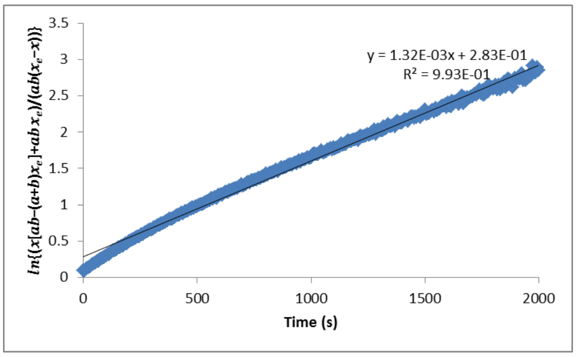
y = 1.32E-03x + 2.83E-01
R² = 9.93E-01
ln{(x[ab−(a+b)xe]+ab xe)/(ab(xe−x))}
Time (s)
0
0.5
1
1.5
2
2.5
3
3.5
0
500
1000
1500
2000

PFN-5/TEMPONE-H system in dichloromethane at 25°C

Plots of $ln\left\{\frac{x[ab-(a+b)x_e]+ab\,x_e}{ab(x_e-x)}\right\}$ versus time, slope = $k_f\left(\frac{2ab-(a+b)x_e}{x_e}\right)$

No base (0 eq TEA)

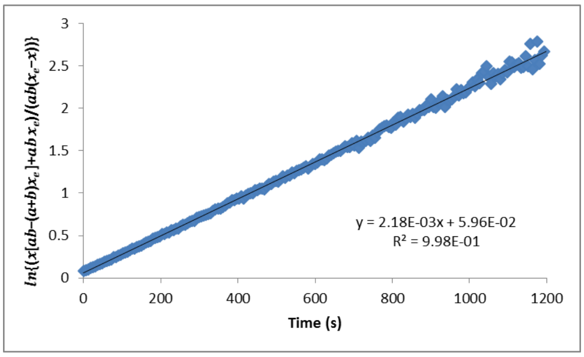

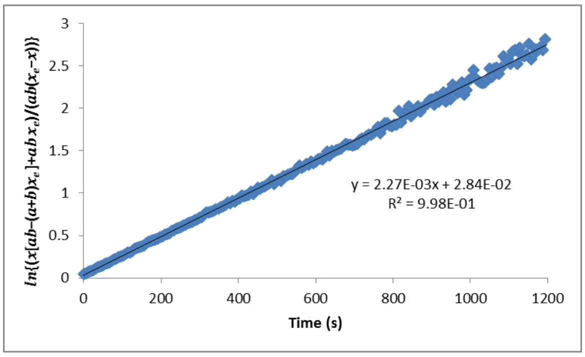

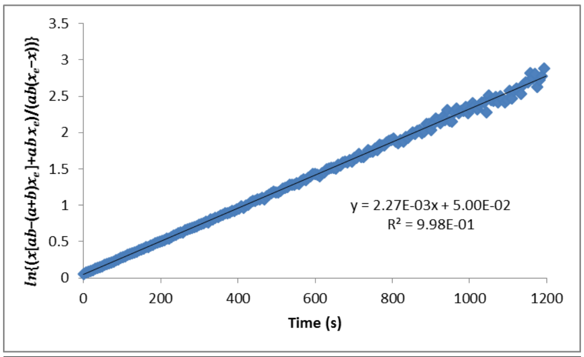

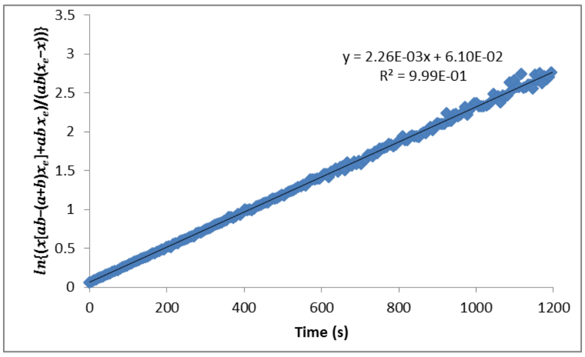

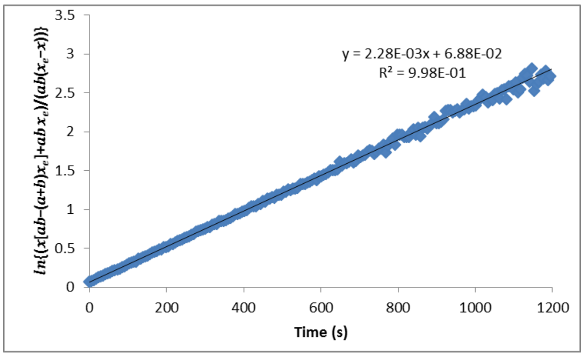

50 eq TEA

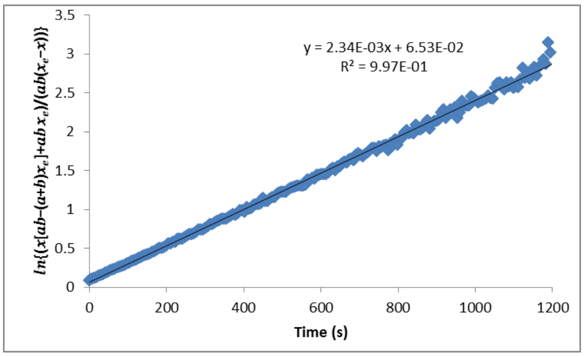

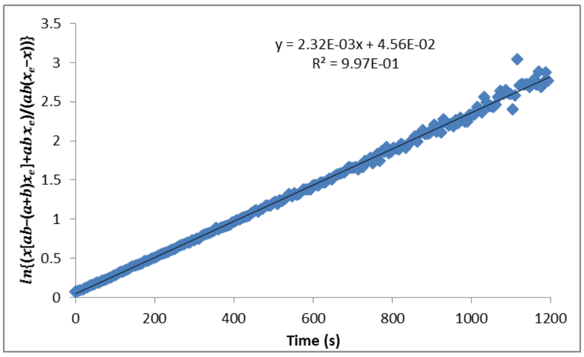

100 eq TEA

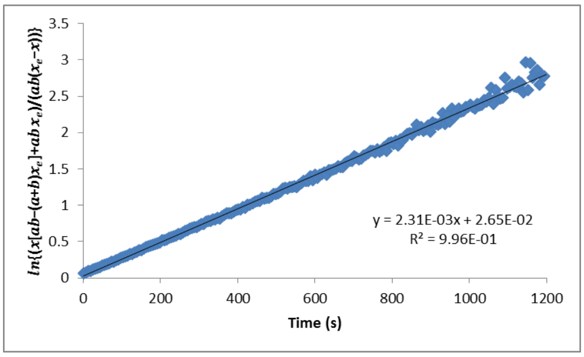

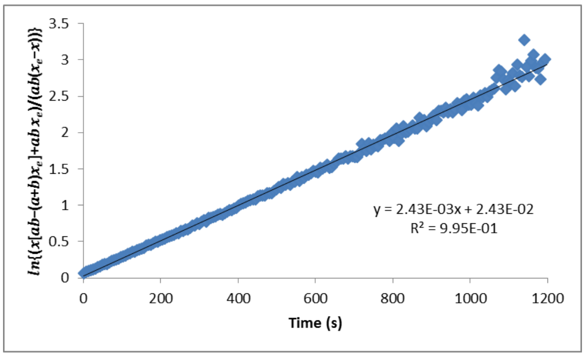

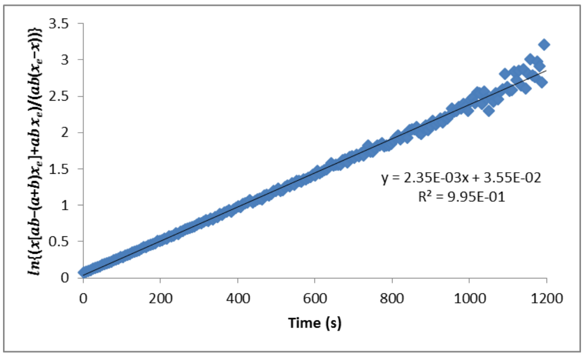

120 eq TEA

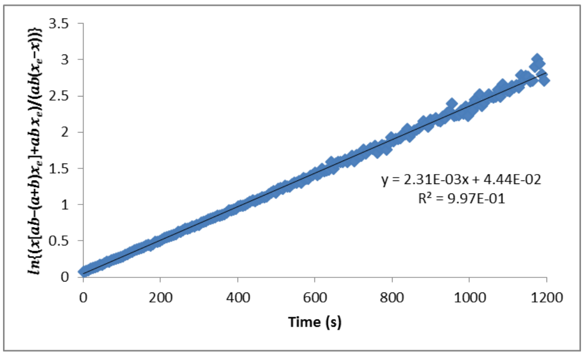

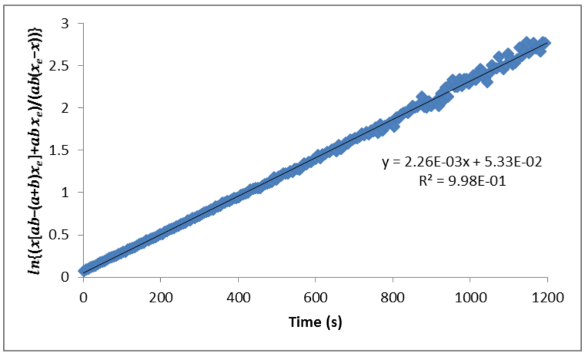

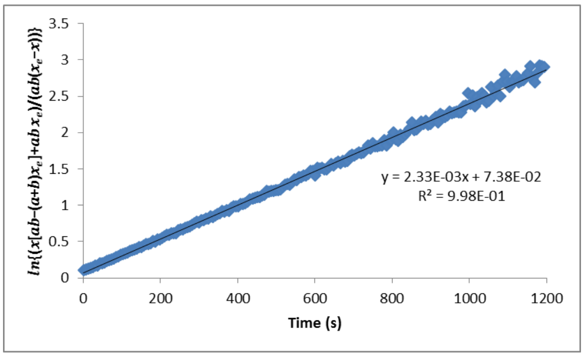
y = 2.33E-03x + 7.38E-02
R² = 9.98E-01
Time (s)

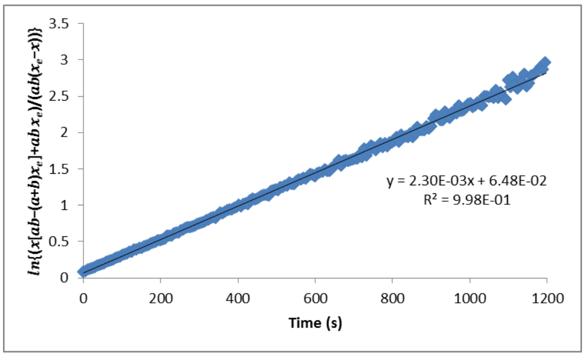
y = 2.30E-03x + 6.48E-02
R² = 9.98E-01
Time (s)

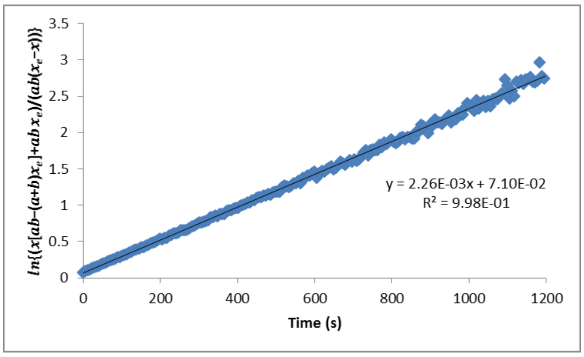
y = 2.26E-03x + 7.10E-02
R² = 9.98E-01
Time (s)

Appendix S5. Kinetic and Thermodynamic Data

Table S1. Exchange Reaction of PFN-*5* and 4-CT-H in dichloromethane at 25°C

	Final Equilibrium concentrations (M)						
**Equivalents of TEA**	**[4-CT-H] M**	**[PFN-*5*] M**	**[4-CT] M**	**[PFN-*5*H] M**	**K**	**k_f (L.mol^{-1}.s^{-1})**	**k_r (L.mol-^{1}.s^{-1})**
**0**	2.52 x 10^{-5}	2.18 x 10^{-5}	9.58 x 10^{-6}	9.58 x 10^{-6}	0.17	6.3	37.8
**0**	2.52 x 10^{-5}	2.18 x 10^{-5}	9.58 x 10^{-6}	9.58 x 10^{-6}	0.17	6.5	39.2
**0**	2.54 x 10^{-5}	2.20 x 10^{-5}	9.39 x 10^{-6}	9.39 x 10^{-6}	0.16	6.2	39.6
**0**	2.53 x 10^{-5}	2.19 x 10^{-5}	9.51 x 10^{-6}	9.51 x 10^{-6}	0.16	6.5	40.0
**10**	1.70 x 10^{-5}	1.37 x 10^{-5}	1.78 x 10^{-5}	1.78 x 10^{-5}	1.36	25.3	18.7
**10**	1.74 x 10^{-5}	1.41 x 10^{-5}	1.73 x 10^{-5}	1.73 x 10^{-5}	1.23	23.3	19.0
**10**	1.59 x 10^{-5}	1.25 x 10^{-5}	1.89 x 10^{-5}	1.89 x 10^{-5}	1.79	32.1	18.0
**10**	1.62 x 10^{-5}	1.28 x 10^{-5}	1.86 x 10^{-5}	1.86 x 10^{-5}	1.67	32.0	19.1
**10**	1.50 x 10^{-5}	1.16 x 10^{-5}	1.96 x 10^{-5}	1.96 x 10^{-5}	2.20	41.4	18.8
**50**	1.32 x 10^{-5}	9.81 x 10^{-6}	2.15 x 10^{-5}	2.15 x 10^{-5}	3.59	70.3	19.6
**50**	1.31 x 10^{-5}	9.75 x 10^{-6}	2.16 x 10^{-5}	2.16 x 10^{-5}	3.66	66.3	18.1
**50**	1.23 x 10^{-5}	8.94 x 10^{-6}	2.24 x 10^{-5}	2.24 x 10^{-5}	4.57	79.9	17.5
**50**	1.28 x 10^{-5}	9.40 x 10^{-6}	2.20 x 10^{-5}	2.20 x 10^{-5}	4.02	80.4	20.0
**75**	1.25 x 10^{-5}	9.18 x 10^{-6}	2.21 x 10^{-5}	2.21 x 10^{-5}	4.27	94.7	22.2
**75**	1.25 x 10^{-5}	9.12 x 10^{-6}	2.22 x 10^{-5}	2.22 x 10^{-5}	4.34	94.3	21.7
**75**	1.18 x 10^{-5}	8.40 x 10^{-6}	2.29 x 10^{-5}	2.29 x 10^{-5}	5.32	110.5	20.8
**75**	1.20 x 10^{-5}	8.66 x 10^{-6}	2.27 x 10^{-5}	2.27 x 10^{-5}	4.94	120.7	24.4
**100**	1.22 x 10^{-5}	8.83 x 10^{-6}	2.25 x 10^{-5}	2.25 x 10^{-5}	4.69	110.1	23.5
**100**	1.22 x 10^{-5}	8.75 x 10^{-6}	2.24 x 10^{-5}	2.24 x 10^{-5}	4.70	110.1	23.4
**100**	1.17 x 10^{-5}	8.35 x 10^{-6}	2.29 x 10^{-5}	2.29 x 10^{-5}	5.39	135.9	25.2
**100**	1.19 x 10^{-5}	8.39 x 10^{-6}	2.28 x 10^{-5}	2.28 x 10^{-5}	5.20	134.0	25.8
**120**	1.28 x 10^{-5}	9.43 x 10^{-6}	2.19 x 10^{-5}	2.19 x 10^{-5}	3.65	121.3	33.2
**120**	1.31 x 10^{-5}	9.74 x 10^{-6}	2.16 x 10^{-5}	2.16 x 10^{-5}	3.97	122.2	30.8
**120**	1.14 x 10^{-5}	8.08 x 10^{-6}	2.32 x 10^{-5}	2.32 x 10^{-5}	5.36	145.2	27.1
**120**	1.17 x 10^{-5}	8.37 x 10^{-6}	2.29 x 10^{-5}	2.29 x 10^{-5}	5.83	146.7	25.2
**150**	1.23 x 10^{-5}	8.91 x 10^{-6}	2.24 x 10^{-5}	2.24 x 10^{-5}	4.58	133.6	29.2

**150**	1.26×10^{-5}	9.21×10^{-6}	2.21×10^{-5}	2.21×10^{-5}	4.21	128.7	30.6
**150**	1.18×10^{-5}	8.43×10^{-6}	2.28×10^{-5}	2.28×10^{-5}	5.25	165.0	31.4
**150**	1.17×10^{-5}	8.34×10^{-6}	2.29×10^{-5}	2.29×10^{-5}	5.39	168.6	31.3
**200**	1.23×10^{-5}	9.00×10^{-6}	2.23×10^{-5}	2.23×10^{-5}	4.47	133.5	29.9
**200**	1.21×10^{-5}	8.79×10^{-6}	2.25×10^{-5}	2.25×10^{-5}	4.73	134.1	28.3
**200**	1.24×10^{-5}	9.04×10^{-6}	2.22×10^{-5}	2.22×10^{-5}	4.41	169.4	38.4
**200**	1.22×10^{-5}	8.84×10^{-6}	2.24×10^{-5}	2.24×10^{-5}	4.67	175.3	37.6

**Number of equivalents of triethylamine relative to the concentration of 4-CT-H*

Table S2. Exchange Reaction of PFN-*5* and 4-CT-H in dichloromethane at 10° C

	Final Equilibrium concentrations (M)						
**Equivalents of TEA***	**[4-CT-H] M**	**[PFN-*5*] M**	**[4-CT] M**	**[PFN-*5*H] M**	**K**	**k_f ($L.mol^{-1}.s^{-1}$)**	**k_r ($L.mol$-$^{1}.s^{-1}$)**
**0**	2.97×10^{-5}	2.34×10^{-5}	5.05×10^{-6}	5.05E-06	0.04	1.2	34.0
**0**	2.92×10^{-5}	2.29×10^{-5}	5.58×10^{-6}	5.58E-06	0.05	1.4	30.6
**120**	1.21×10^{-5}	5.81×10^{-6}	2.25×10^{-5}	2.25E-05	7.18	100.8	14.0
**120**	1.23×10^{-5}	5.95×10^{-6}	2.23×10^{-5}	2.23E-05	6.84	96.5	14.1
**200**	1.14×10^{-5}	5.07×10^{-6}	2.31×10^{-5}	2.31E-05	9.28	133.1	14.3
**200**	1.15×10^{-5}	5.15×10^{-6}	2.30×10^{-5}	2.30E-05	9.00	130.7	14.5

**Number of equivalents of triethylamine relative to the concentration of 4-CT-H*

Table S3. Exchange Reaction of PFN-*5* and 4-CT-H in acetonitrile at 25°C

		Final Equilibrium Concentrations (M)					
**Eq TEA***	**[PFN-*5*]**	**4-CT-H**	**[PFN-*5*H]**	**[4-CT]**	**K**	k_f **(L.mol^{-1}.s^{-1})**	k_r **(L.mol^{-1}.s^{-1})**
**0**	1.32×10^{-5}	1.32×10^{-5}	1.67×10^{-5}	1.67×10^{-5}	1.60	25.9	16.2
**0**	1.47×10^{-5}	1.47×10^{-5}	1.51×10^{-5}	1.51×10^{-5}	1.06	20.7	19.6
**0**	1.58×10^{-5}	1.58×10^{-5}	1.40×10^{-5}	1.40×10^{-5}	0.78	17.8	22.8
**0**	1.63×10^{-5}	1.63×10^{-5}	1.35×10^{-5}	1.35×10^{-5}	0.68	16.8	24.6
**0**	1.69×10^{-5}	1.53×10^{-5}	1.45×10^{-5}	1.45×10^{-5}	0.81	19.3	23.8
**0**	1.58×10^{-5}	1.43×10^{-5}	1.54×10^{-5}	1.54×10^{-5}	1.05	23.4	22.3
**50**	1.35×10^{-5}	1.35×10^{-5}	1.63×10^{-5}	1.63×10^{-5}	1.46	25.2	17.3
**50**	1.40×10^{-5}	1.40×10^{-5}	1.58×10^{-5}	1.58×10^{-5}	1.28	23.8	18.6
**50**	1.32×10^{-5}	1.32×10^{-5}	1.67×10^{-5}	1.67×10^{-5}	1.08	34.6	32.1
**50**	1.65×10^{-5}	1.49×10^{-5}	1.49×10^{-5}	1.49×10^{-5}	0.90	21.5	23.9
**75**	1.50×10^{-5}	1.49×10^{-5}	1.48×10^{-5}	1.48×10^{-5}	0.98	20.8	21.3
**75**	1.46×10^{-5}	1.46×10^{-5}	1.52×10^{-5}	1.52×10^{-5}	1.08	21.7	20.1
**75**	1.65×10^{-5}	1.50×10^{-5}	1.47×10^{-5}	1.47×10^{-5}	0.87	21.9	25.1
**75**	1.68×10^{-5}	1.53×10^{-5}	1.45×10^{-5}	1.45×10^{-5}	0.81	19.9	24.4
**120**	1.14×10^{-5}	1.14×10^{-5}	1.83×10^{-5}	1.83×10^{-5}	2.56	35.4	13.8
**120**	1.32×10^{-5}	1.32×10^{-5}	1.67×10^{-5}	1.67×10^{-5}	2.37	32.8	13.8
**120**	1.60×10^{-5}	1.45×10^{-5}	1.53×10^{-5}	1.53×10^{-5}	1.00	22.4	22.4
**120**	1.72×10^{-5}	1.57×10^{-5}	1.41×10^{-5}	1.41×10^{-5}	0.74	18.3	24.7

**Number of equivalents of triethylamine relative to the concentration of 4-CT-H*

Table S4. Exchange reaction of PFN-*5* and TEMPONE-H in dichloromethane at 25° C

	Final Equilibrium Concentrations (M)						
**Equivalents of TEA***	**[TEMPONE-H]**	**[PFN-*5*]**	**[TEMPONE]**	**[PFN-5H]**	**K**	k_f **(L.mol^{-1}.s^{-1})**	k_r **(L.mol-^{1}.s^{-1})**
**0**	1.55×10^{-5}	1.34×10^{-5}	1.95×10^{-5}	1.95×10^{-5}	1.84	43.5	23.6
**0**	1.60×10^{-5}	1.39×10^{-5}	1.90×10^{-5}	1.90×10^{-5}	1.62	42.6	26.2
**0**	1.66×10^{-5}	1.45×10^{-5}	1.84×10^{-5}	1.84×10^{-5}	1.41	40.8	29.0
**0**	1.71×10^{-5}	1.49×10^{-5}	1.80×10^{-5}	1.80×10^{-5}	1.26	37.4	29.6
**0**	1.73×10^{-5}	1.51×10^{-5}	1.78×10^{-5}	1.78×10^{-5}	1.21	36.9	30.5
**50**	1.61×10^{-5}	1.39×10^{-5}	1.90×10^{-5}	1.90×10^{-5}	1.61	43.6	27.2
**50**	1.61×10^{-5}	1.40×10^{-5}	1.89×10^{-5}	1.89×10^{-5}	1.59	43.1	27.1
**100**	1.58×10^{-5}	1.36×10^{-5}	1.92×10^{-5}	1.92×10^{-5}	1.71	44.6	26.1
**100**	1.66×10^{-5}	1.45×10^{-5}	1.83×10^{-5}	1.83×10^{-5}	1.40	42.4	30.3
**100**	1.74×10^{-5}	1.53×10^{-5}	1.75×10^{-5}	1.75×10^{-5}	1.14	37.1	32.5
**120**	1.63×10^{-5}	1.42×10^{-5}	1.86×10^{-5}	1.86×10^{-5}	1.50	41.8	27.8
**120**	1.60×10^{-5}	1.42×10^{-5}	1.86×10^{-5}	1.86×10^{-5}	1.52	41.4	27.2
**120**	1.72×10^{-5}	1.54×10^{-5}	1.74×10^{-5}	1.74×10^{-5}	1.14	36.9	32.4
**120**	1.72×10^{-5}	1.54×10^{-5}	1.74×10^{-5}	1.74×10^{-5}	1.15	36.6	31.9
**120**	1.73×10^{-5}	1.55×10^{-5}	1.72×10^{-5}	1.72×10^{-5}	1.10	35.2	32.0

**Number of equivalents of triethylamine relative to the concentration of TEMPONE-H*

Appendix S6. NMR Spectra of Synthesised Compounds

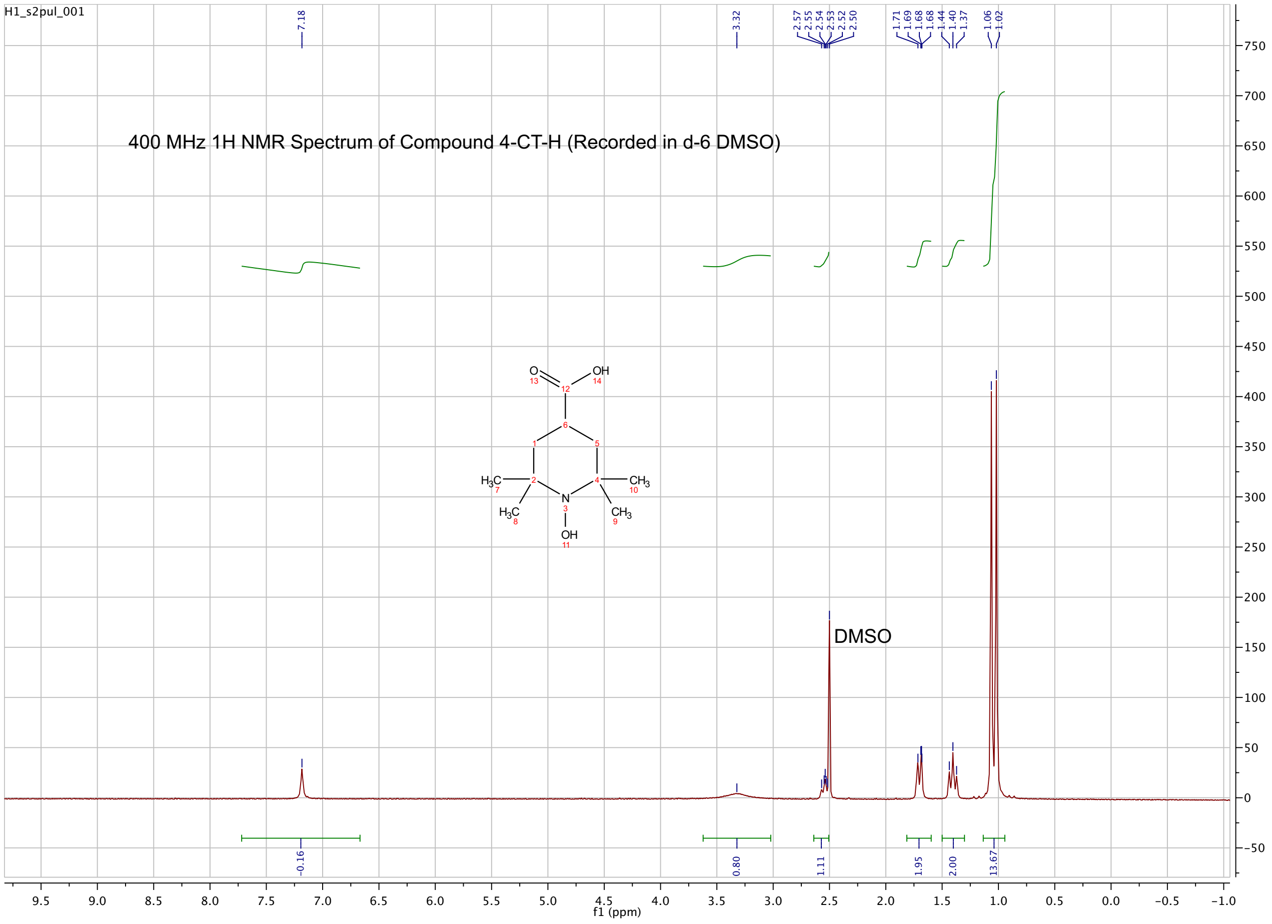

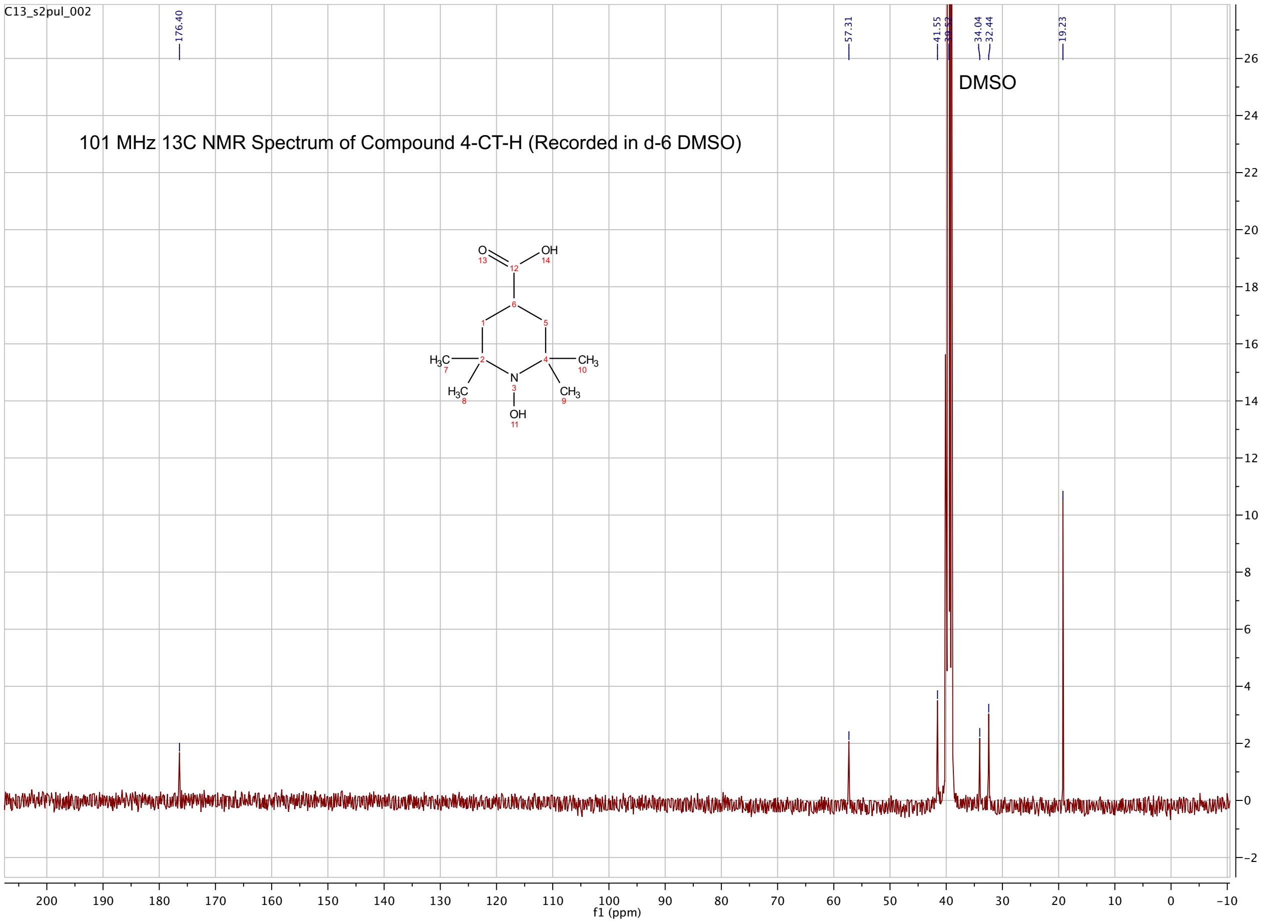
C13_s2pul_002
176.40
57.31
41.55
39.52
34.04
32.44
19.23
DMSO
101 MHz 13C NMR Spectrum of Compound 4-CT-H (Recorded in d-6 DMSO)
f1 (ppm)

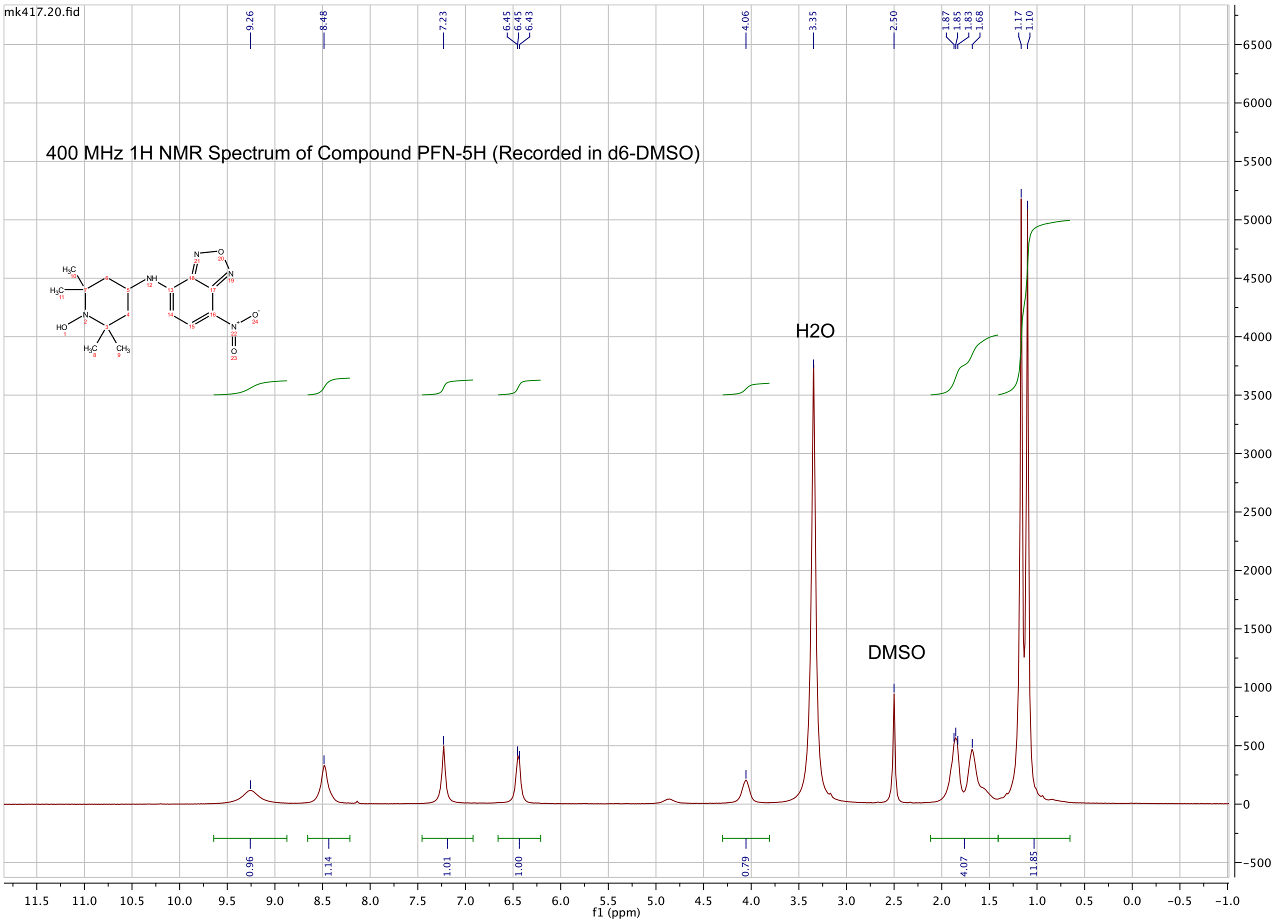

mk417.20.fid
400 MHz 1H NMR Spectrum of Compound PFN-5H (Recorded in d6-DMSO)
H2O
DMSO
9.26
8.48
7.23
6.45
6.45
6.43
4.06
3.35
2.50
1.87
1.85
1.83
1.68
1.17
1.10
0.96
1.14
1.01
1.00
0.79
4.07
11.85
f1 (ppm)

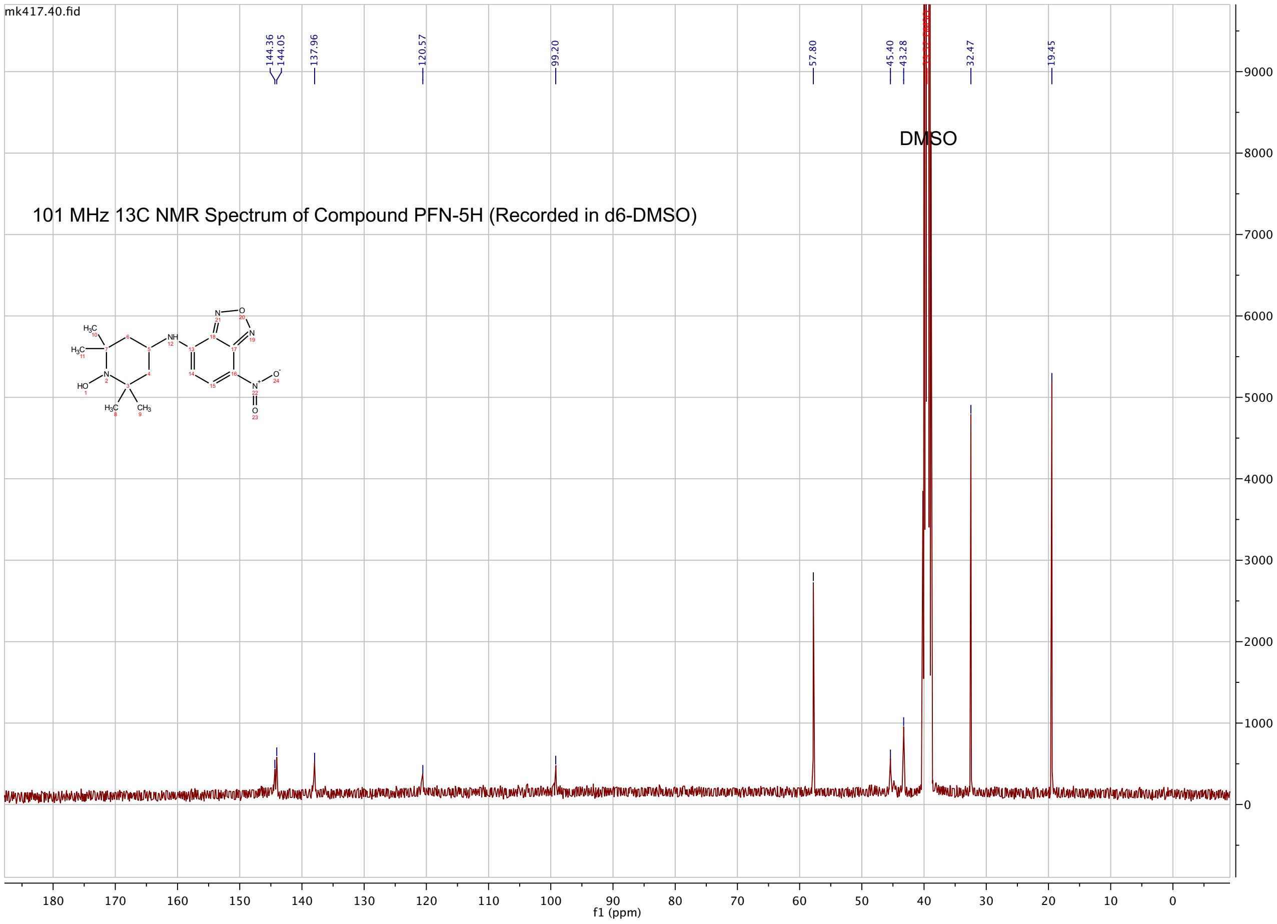
mk417.40.fid
144.36
144.05
137.96
120.57
99.20
57.80
45.40
43.28
32.47
19.45
DMSO
101 MHz 13C NMR Spectrum of Compound PFN-5H (Recorded in d6-DMSO)
f1 (ppm)

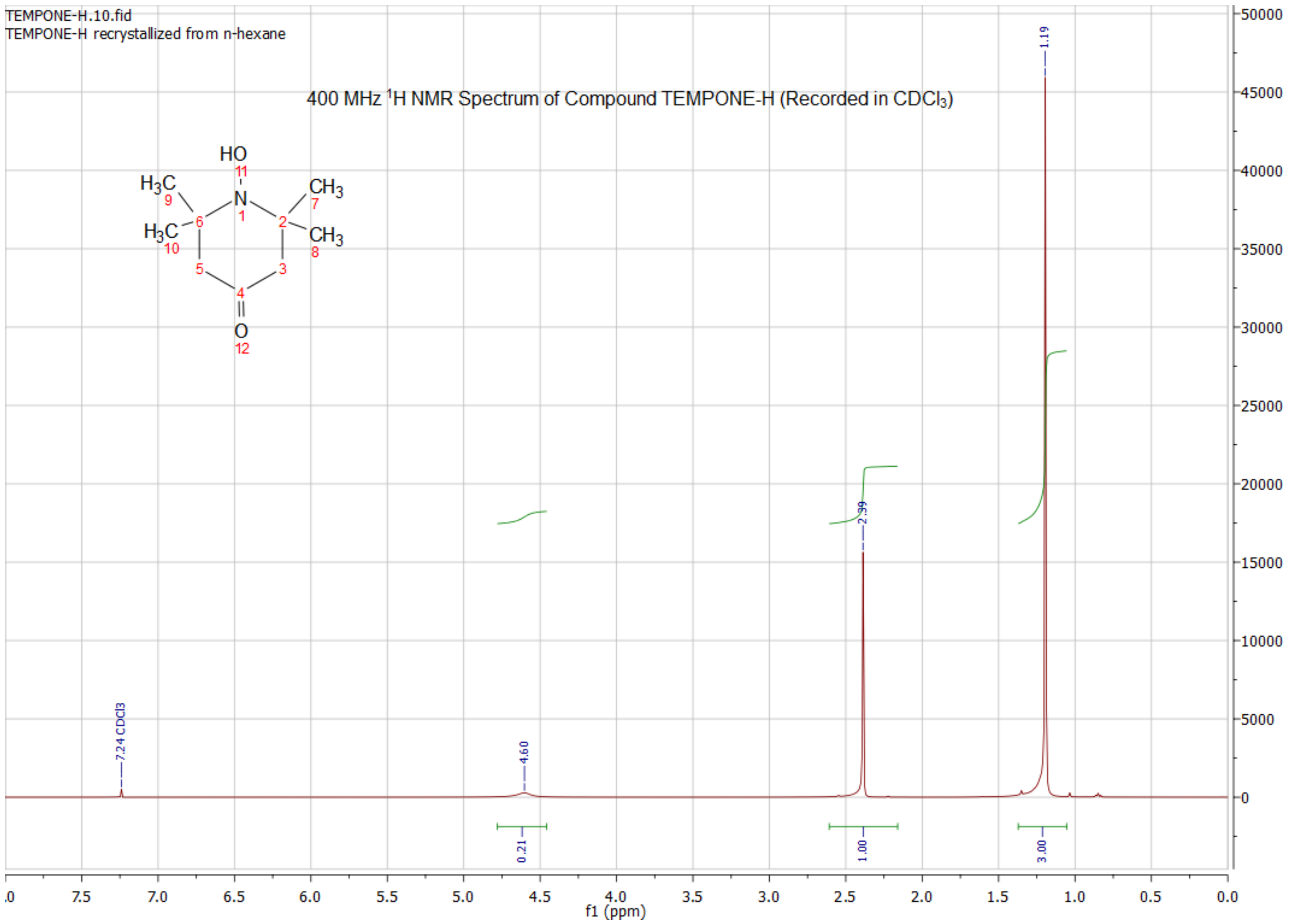

TEMPONE-H.10.fid
TEMPONE-H recrystallized from n-hexane
400 MHz 1H NMR Spectrum of Compound TEMPONE-H (Recorded in CDCl3)
HO
N
H3C
H3C
CH3
CH3
O
7.24 CDCl3
4.60
2.39
1.19
0.21
1.00
3.00
f1 (ppm)

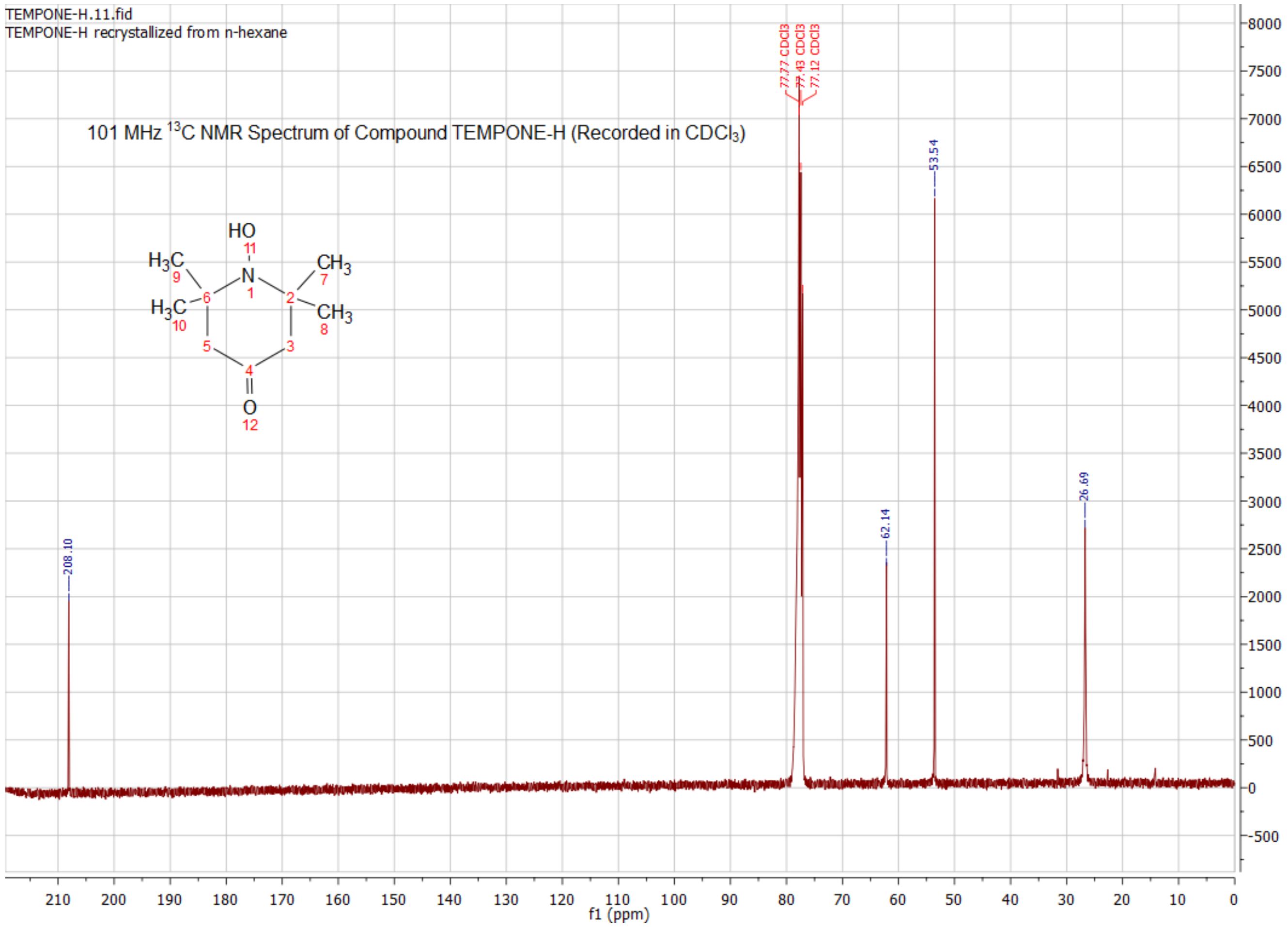

TEMPONE-H.11.fid
TEMPONE-H recrystallized from n-hexane
101 MHz ¹³C NMR Spectrum of Compound TEMPONE-H (Recorded in CDCl₃)
HO
N
H3C
CH3
O
77.77 CDCl3
77.43 CDCl3
77.12 CDCl3
208.10
62.14
53.54
26.69
f1 (ppm)

Appendix S7. References

1. M. J. Jones, G. Moad, E. Rizzardo, and D. H. Solomon, *J. Org. Chem*. 1989 **54**, 1607-1611.
2. M. Becker, L. De Cola, and A. Studer, *Chem. Commun*. 2011 **47**, 3392-3394.
